# Three in one: evolution of viviparity, coenocytic placenta and polyembryony in cyclostome bryozoans

**DOI:** 10.1186/s12862-021-01775-z

**Published:** 2021-04-12

**Authors:** U. A. Nekliudova, T. F. Schwaha, O. N. Kotenko, D. Gruber, N. Cyran, A. N. Ostrovsky

**Affiliations:** 1 Department of Evolutionary Biology, Integrative Zoology, Faculty of Life Sciences, University of Vienna, Althanstr. 14, 1090 Vienna, Austria; 2 Department of Invertebrate Zoology, Faculty of Biology, Saint Petersburg State University, Universitetskaja nab. 7/9, 199034 Saint Petersburg, Russia; 3 Core Facility Cell Imaging and Ultrastructure Research, Faculty of Life Sciences, University of Vienna, Althanstr. 14, 1090 Vienna, Austria; 4 Department of Palaeontology, Faculty of Earth Sciences, Geography and Astronomy, University of Vienna, Althanstr. 14, 1090 Vienna, Austria

**Keywords:** Viviparity, Placenta, Coenocyte, Polyembryony, Evolution, Colonial invertebrates

## Abstract

**Background:**

Placentation has evolved multiple times among both chordates and invertebrates. Although they are structurally less complex, invertebrate placentae are much more diverse in their origin, development and position. Aquatic colonial suspension-feeders from the phylum Bryozoa acquired placental analogues multiple times, representing an outstanding example of their structural diversity and evolution. Among them, the clade Cyclostomata is the only one in which placentation is associated with viviparity and polyembryony—a unique combination not present in any other invertebrate group.

**Results:**

The histological and ultrastructural study of the sexual polymorphic zooids (gonozooids) in two cyclostome species, *Crisia eburnea* and *Crisiella producta*, revealed embryos embedded in a placental analogue (nutritive tissue) with a unique structure—comprising coenocytes and solitary cells—previously unknown in animals. Coenocytes originate via nuclear multiplication and cytoplasmic growth among the cells surrounding the early embryo. This process also affects cells of the membranous sac, which initially serves as a hydrostatic system but later becomes main part of the placenta. The nutritive tissue is both highly dynamic, permanently rearranging its structure, and highly integrated with its coenocytic ‘elements’ being interconnected via cytoplasmic bridges and various cell contacts. This tissue shows evidence of both nutrient synthesis and transport (bidirectional transcytosis), supporting the enclosed multiple progeny. Growing primary embryo produces secondary embryos (via fission) that develop into larvae; both the secondary embyos and larvae show signs of endocytosis. Interzooidal communication pores are occupied by 1‒2 specialized pore-cells probably involved in the transport of nutrients between zooids.

**Conclusions:**

Cyclostome nutritive tissue is currently the only known example of a coenocytic placental analogue, although syncytial ‘elements’ could potentially be formed in them too. Structurally and functionally (but not developmentally) the nutritive tissue can be compared with the syncytial placental analogues of certain invertebrates and chordates. Evolution of the cyclostome placenta, involving transformation of the hydrostatic apparatus (membranous sac) and change of its function to embryonic nourishment, is an example of exaptation that is rather widespread among matrotrophic bryozoans. We speculate that the acquisition of a highly advanced placenta providing massive nourishment might support the evolution of polyembryony in cyclostomes. In turn, massive and continuous embryonic production led to the evolution of enlarged incubating polymorphic gonozooids hosting multiple progeny.

## Background

Evolution of parental care is a key novelty affecting offspring fitness and survival, influencing the life history mode and, ultimately, governing the evolutionary success of the particular group of organisms [1–3]. One of the most widespread modes of parental care is the retention of developing progeny inside the parental body. This provides protection and is accompanied by an exchange of gases eventually triggering a bidirectional transport of metabolites between them. The final step is the acquisition of matrotrophy (extraembryonic nutrition, EEN)—direct extra-vitelline provisioning of nutrients to the progeny during incubation [4–7]. This mode is widespread among Animalia, proven or inferred in 22 of 34 phyla, and independently originated between at least 140 and 145 times. In general, the matrotrophic adaptations of invertebrates are anatomically simpler than those of chordates, but demonstrate a higher positional, structural and functional diversity [7, 8].

The most complex matrotrophic mode is placentotrophy, and its repeated origins in different chordate and invertebrate clades (altogether in 16 phyla) indicate the evolutionary effectiveness of this strategy [4, 6, 7, 9]. Its benefits include saving the energy required for yolk production, reducing the number of eggs, as well as creating and maintaining a comfortable environment for developing offspring, resulting in their greater fitness [4, 5]. According to Mossman ([10], p. 156), a placenta is “any intimate apposition or fusion of the fetal organs to the maternal tissues for physiological exchange”. Whereas vertebrate placentae are mostly (although not always) of a similar origin involving sexual ducts and embryonic envelopes (reviewed in [4, 6, 9, 11–14]), the placental analogues of invertebrates originated from a plethora of tissues and organs and sometimes involve embryonic envelopes as well. Generally, a ‘placental analogue’ is any local zone of enhanced nutritional transport developing during incubation, from simple apposition of non-specialized epithelia to specialized parental–embryonic tissue/cell complexes that increase the entire secreting and absorbing surfaces. In many instances, the specialized nutritive structures are formed either by the parent or by the embryo alone, yet forming a bilateral interface providing a bidirectional transfer of nutrients (reviewed in [7]; see also [15]).

The phylum Bryozoa, which are widespread sedentary colonial suspension-feeders, exhibits the largest proportion of placental species among aquatic invertebrates [7, 16]. Among three bryozoan classes, all phylactolaemates, all extant stenolaemates and many gymnolaemates exhibit placentation. This evolved independently once in both the Phylactolaemata and Stenolaemata, and repeatedly in Gymnolaemata [7, 17–21]. Their placental analogues therefore differ in position, structure and origin, making this phylum an exceptional group for comparative evolutionary studies. Only a few detailed works on bryozoan placentation are available, all of which focus on gymnolaemates [15, 22–26].

The order Cyclostomata encompasses living representatives of the class Stenolaemata, one of the most ancient and successful bryozoan clades that has been documented since the early Ordovician and remains quite diverse and abundant in modern marine benthic communities [27–29]. One of the factors potentially contributing to stenolaemate success is embryonic incubation, which has evolved one or possibly more times in their history in the Paleozoic and Mesozoic (discussed in [16, 30–33]). Cyclostome incubation chambers (gonozooids) first appear in the fossil record in the late Triassic and since then are known in all except one cyclostome family [34, 35].

Reproduction of recent cyclostome bryozoans is remarkable in combining viviparity (intracoelomic incubation), placentation and polyembryony [36]. This unique reproductive pattern may have played an important role in their past and current success, making this group a promising model system for studying the evolution of complex reproductive traits and their significance. Our understanding of the reproductive biology of Cyclostomata, however, is hampered by a severe lack of data (stressed by [36]). Indeed, apart from the research on sperm morphology and ultrastructure [37–39], only five early publications focused on cyclostome oogenesis and embryonic incubation [40–44]. Several other papers include additional information [31, 45–54], but these data are rather fragmentary. Although the first descriptions of cyclostome embryos and larvae were published more than 150 years ago [55–57], only a few papers have dealt with the embryonic development, larval structure and metamorphosis of Cyclostomata since then [31, 58–65]. The most detailed piece of work including all aspects of cyclostome reproduction from spermiogenesis to larval structure is a monograph by Borg [44], which still remains the main source of information on this topic. All aforementioned studies were based on histological techniques that, considering the small size of most cyclostomes, led to many gaps in our knowledge as well as incorrect interpretations of the morphology and dubious or contradictory statements. Only two ultrastructural studies of cyclostome oogenesis and embryogenesis (non-published diploma work by Dolinina [66]), and larval microanatomy [67] are known. Recently, Nielsen with co-authors [68] studied microanatomy of the cyclostome larva and ancestrula using immunochemistry and confocal laser microscopy.

**Fig. 1:**
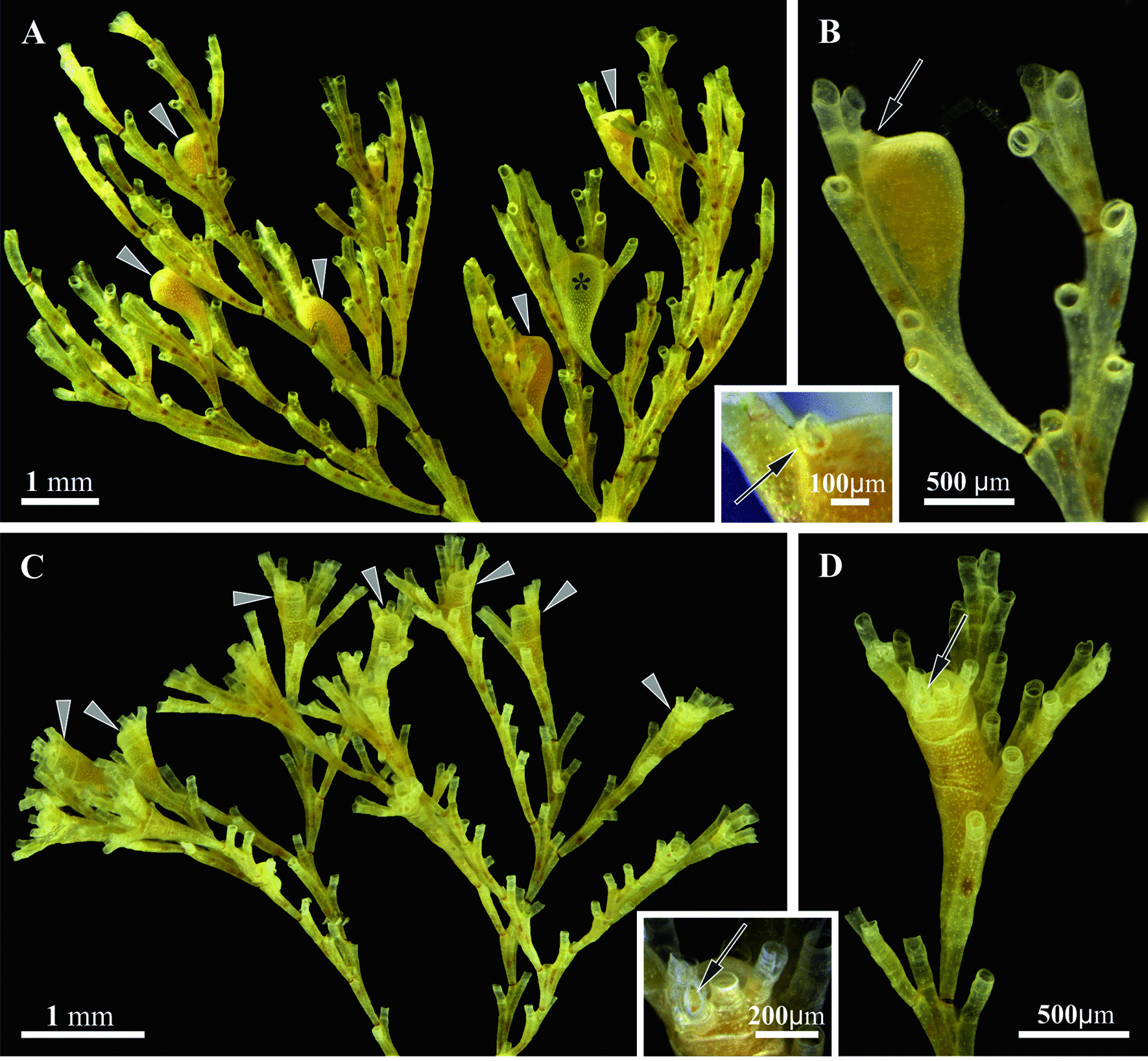
General view of mature colonies and their details (stereomicroscope). **a, b**, insert, *Crisia eburnea*. **a** Mature colony having six gonozooids, mostly with embryos (arrowheads) and one empty (asterisk); **b** Ramifying branch with gonozooid (arrow: ooeciopore); insert, distal part of gonozooid showing flattened ooeciopore (arrow). **c, d**, insert, *Crisiella producta*. **c** Mature colony with at least seven gonozooids (arrowheads). **d** Distal tip of the branch with gonozooid (arrow: ooeciopore); insert, Distal part of gonozooid showing flattened ooeciopore (arrow)

**Fig. 2:**
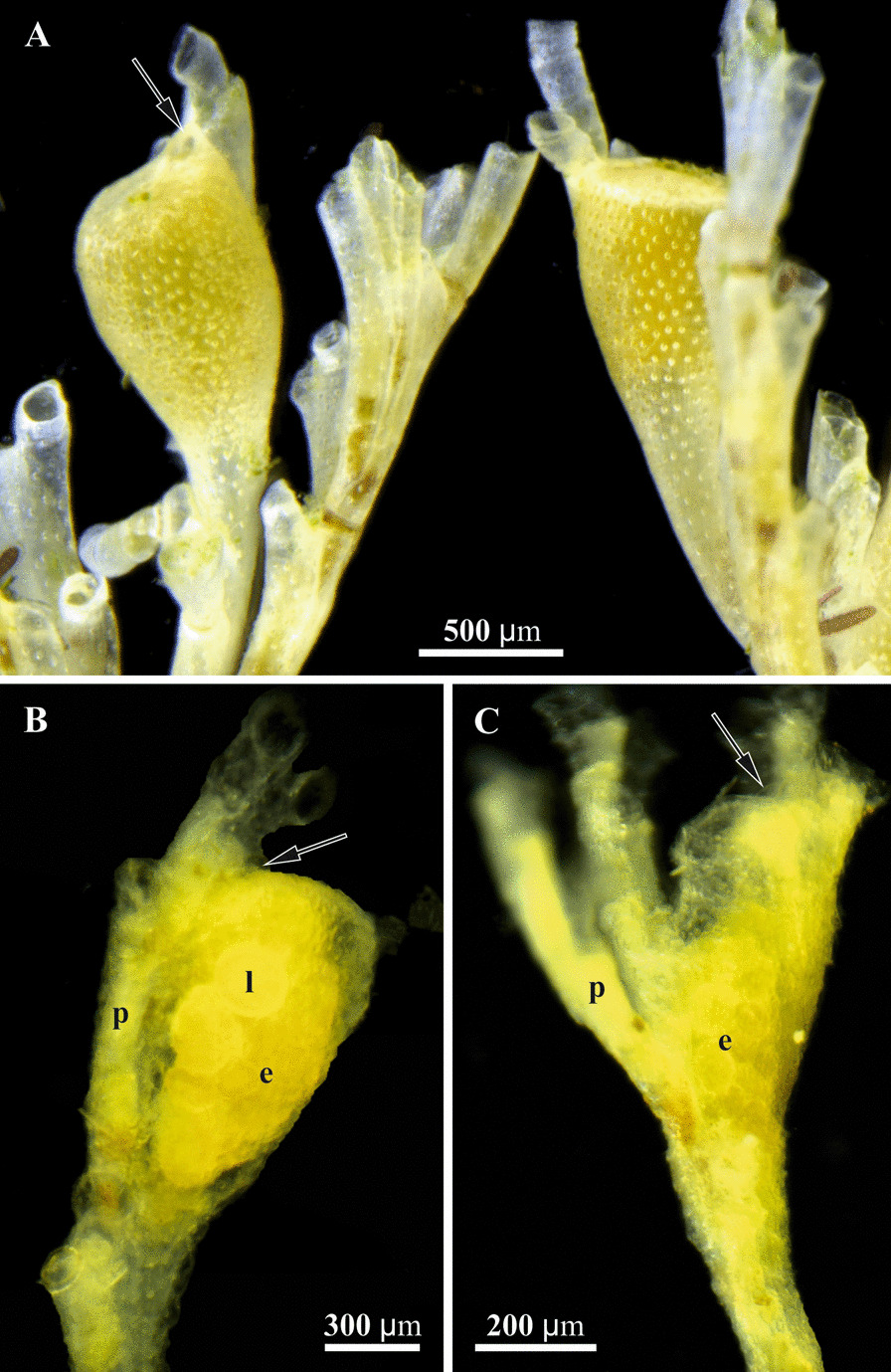
Details of gonozooids (stereomicroscope). **a, b**, *Crisia eburnea.* **a** distal tips of the branches with fully-formed (left) and forming (right) gonozooids (both containing embryos). **b** Decalcified tip of the branch with gonozooid showing embryos and larvae visible through semitransparent zooidal wall. **c** Decalcified tip of the branch with gonozooid containing embryos in *Crisiella producta.* Ooeciostomes shown by arrows. e, embryos; l, larva; p, polypide of autozooid

One of the most intriguing aspects of cyclostome sexual reproduction is the evolutionary transformation of the feeding module (autozooid) to the enlarged non-feeding polymorph (gonozooid), which incubates numerous embryos and larvae (Figs. 1, 2). Except for one known example, cyclostomes are colonial hermaphrodites with either zooidal gonochory or hermaphroditism and pronounced sexual zooidal polymorphism [16, 20, 36, 69]. Sperm is produced in autozooids, whereas female zooids (either autozooids or autozooidal polymorphs possessing a polypide—a tentacle crown associated with a gut) form an ovary, degenerate their polypide and become gonozooids. They greatly exceed autozooids in size and have a modified morphology and anatomy, sometimes merging to form a common incubation chamber. Embryonic development involves polyembryony when a single zygote develops to the primary embryo, which produces numerous (up to 115, [40]) secondary embryos by fission. As a result, each gonozooid produces multiple, genetically identical larvae [70]. Extraembryonic nourishment is provided by the nutritive tissue—a placental analogue of uncertain structure and origin. Apart from the aforementioned, many key aspects of gonozooid development and functioning as well as embryogenesis remain unstudied. Among them are the source and developmental stages of the nutritive tissue, nutrient transport from the autozooids to the gonozooid and from the placenta to the embryos, the formation of the primary embryo, mechanisms of polyembryony and larval development. These major gaps in our knowledge result in a number of inaccuracies and even mistakes that have been continuously repeated in the textbooks (see. e.g. [36, 71, 72]).

**Fig. 3:**
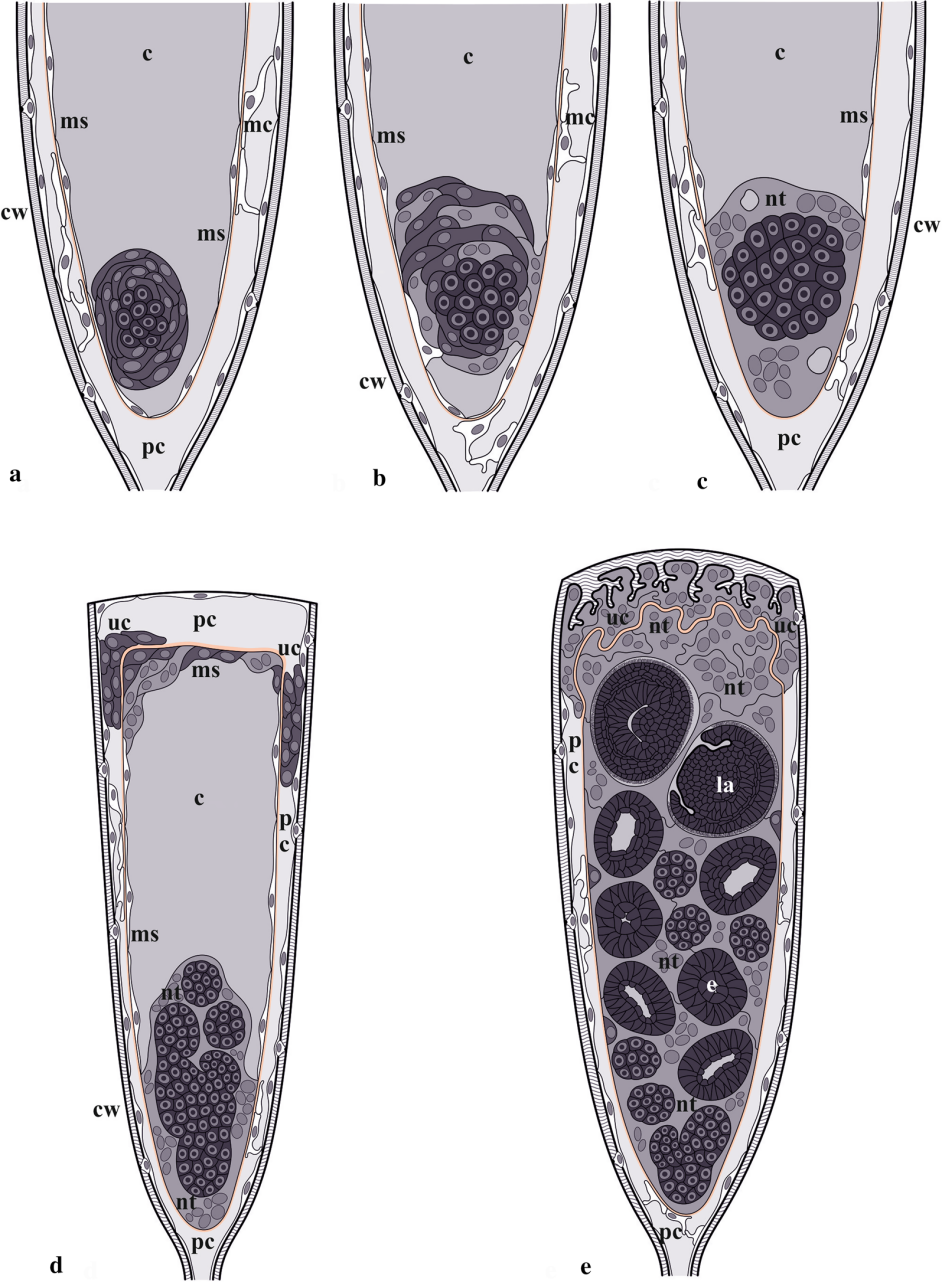
Schematic representations of the gonozooid, nutritive structures and embryonic development in crisiid cyclostomes. **a** Early primary embryo surrounded by multilayered ‘envelope’ inside of non-altered membranous sac (corresponding to Figs. 4b, c, 7a, b); **b** Formation of coenocytic elements inside and outside of the multilayered ‘envelope’; membranous sac wall is partially incorporated into the developing nutritive tissue (corresponding to Figs. 4d, 7c‒e). **c** Early nutritive tissue (corresponding to Figs. 4e, 5a, insert) (in **a**‒**c** distal part of growing gonozooid is not depicted). **d** Primary embryo producing secondary embryos via fission inside growing nutritive tissue (corresponding to Figs. 4f, 5b, insert, c); developing coenocytic elements of the membranous sac roof are surrounded by the ‘upper cell complex’ (see Fig. 7f). **e** Fully-formed gonozooid filled with secondary embryos and young larvae embedded into the nutritive tissue (corresponding to Figs. 4g, H, 6i, 9i, j); dark solitary cells are visible on the periphery of the nutritive tissue (see Fig. 9g, h). In all images, basal lamina shown by red line. c, coelom; cw, cystid wall; e, embryo; la, larva; mc, ‘mesothelial’ cells; ms, membranous sac; nt, nutritive tissue; pc, pseudocoel; uc, ‘upper cell complex’

**Fig. 4:**
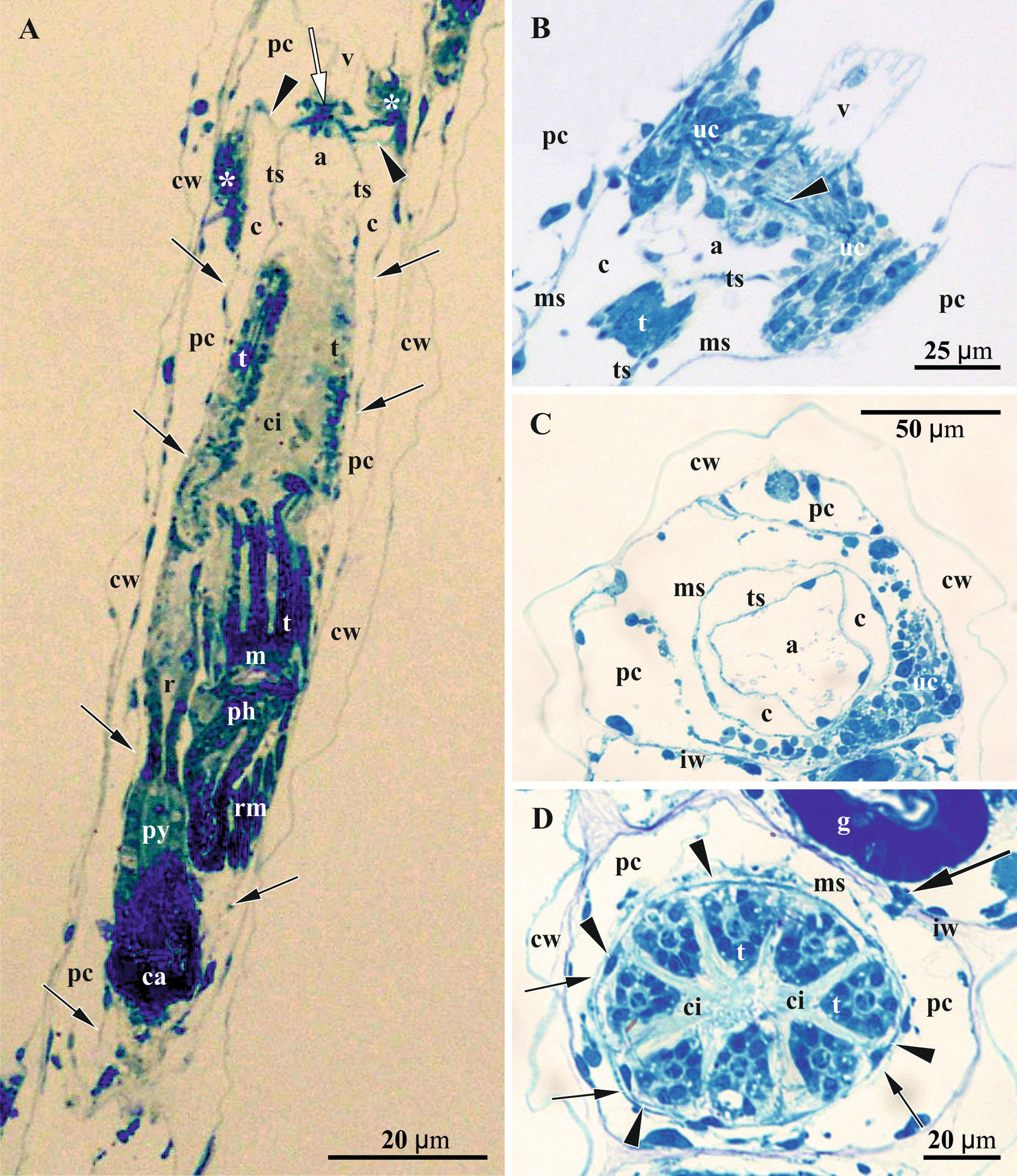
Details of autozooids in *Crisiella producta* (light microscopy). **a** Longitudinal section of autozooid with functional polypide (atrial sphincter shown by white arrow, ‘upper cell complex’ by asterisks, attachment organ by arrowheads, and walls of membranous sac by black arrows). **b** Longitudinal section of distal tip of autozooid showing wrinkled lower part of vestibulum, ‘upper cell complex’ around atrial sphincter (one of its muscles shown by arrowhead) and upper part of the tentacle sheath and membranous sac. **c** Cross-section of autozooid at the level of atrium. **d** Cross-section of autozooid at the level of tentacle crown; membranous sac (smaller arrows) surrounds the tentacle sheath (arrowheads); plugged communication pore in interzooidal wall shown by larger arrow. a, atrium; c, coelom; ca, caecum; ci, tentacle ciliature; cw, external cystid wall; g, gut; iw, interzooidal wall; m, mouth; ms, membranous sac; pc, pseudocoel; ph, pharynx; py, pylorus; r, rectum; rm, retractor muscles; t, tentacle; ts, tentacle sheath; uc, ‘upper cell complex’; v, vestibulum

To fill these gaps and shed light on the morpho-functional transformation of the feeding module to the incubation chamber, we conducted detailed ultrastructural and microanatomical research of gonozooids in two species from the cyclostome family Crisiidae (Figs. 1, 2). The focus was on the process of embryonic incubation, the structure and functioning of the nutritive tissue at its different developmental stages, embryogenesis and polyembryony (Fig. 3). For comparative purposes we additionally studied autozooid anatomy (Fig. 4) (see also [44, 73] and review [74]). In a wider context, the current study compares placentation in different bryozoan lineages and discusses placental ‘syncytia’ in both invertebrates and chordates. We also address the hypothetical consequences of placenta evolution such as polyembryony and polymorphism, and reconstruct hypothetical scenarios for the evolution of sexual reproduction in Cyclostomata.

## Results

### Autozooid

**Fig. 5:**
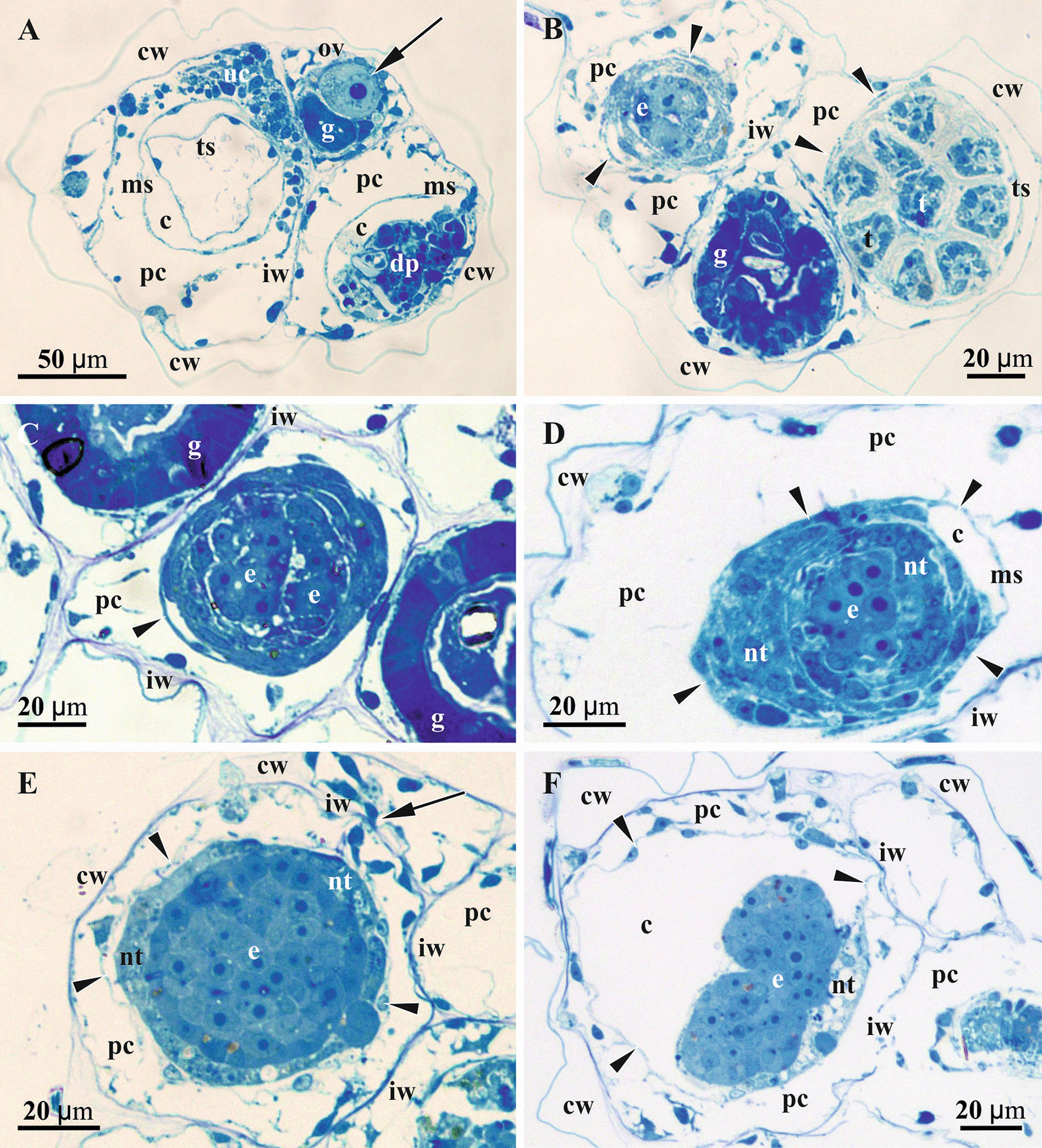
Details of oogenesis and early embryogenesis in *Crisiella producta* (**a**‒**f** Colony branches showing autozooids cross-sectioned on different levels and gonozooids sectioned in their lower part) (light microscopy). **a** Two autozooids and gonozooid containing ovary with mature oligolecithal oocyte (arrow). **b**‒**f** Consecutive stages of embryonic growth and placental development. **b** Gonozooid (upper left) with early primary embryo surrounded by massive multilayered ‘envelope’ presumably originated from follicular cells. **c** Gonozooid with early primary embryo showing loose arrangement of blastomeres and surrounded by multilayered ‘envelope’. **d** Gonozooid with early primary embryo surrounded by multilayered ‘envelope’ transforming to nutritive tissue. **e** Gonozooid with primary embryo surrounded by placental analogue (nutritive tissue). Non-altered membranous sac wall is still visible as a thin ‘nuclei-bearing’ line (arrowheads) either adjacent to or somewhat removed from the multilayered ‘envelope’ or nutritive tissue in **b**‒**e**; plugged communication pore in interzooidal wall shown by arrow in **e**. **f** Gonozooid with late primary embryo starting fission; membranous sac (arrowheads) is partially free and partially incorporated into nutritive tissue. In all gonozooids the pseudocoel (space between placental analogue/membranous sac and cystid wall) is filled with a network of large and irregular, darkly-stained ‘mesothelial’ cells. c, coelom; cw, external cystid wall; dp, degenerating polypide; e, primary embryo; g, gut; iw, interzooidal wall; ms, membranous sac; nt, nutritive tissue; ov, ovary; pc, pseudocoel; t, tentacle; ts, tentacle sheath; uc, ‘upper cell complex’

**Fig. 6:**
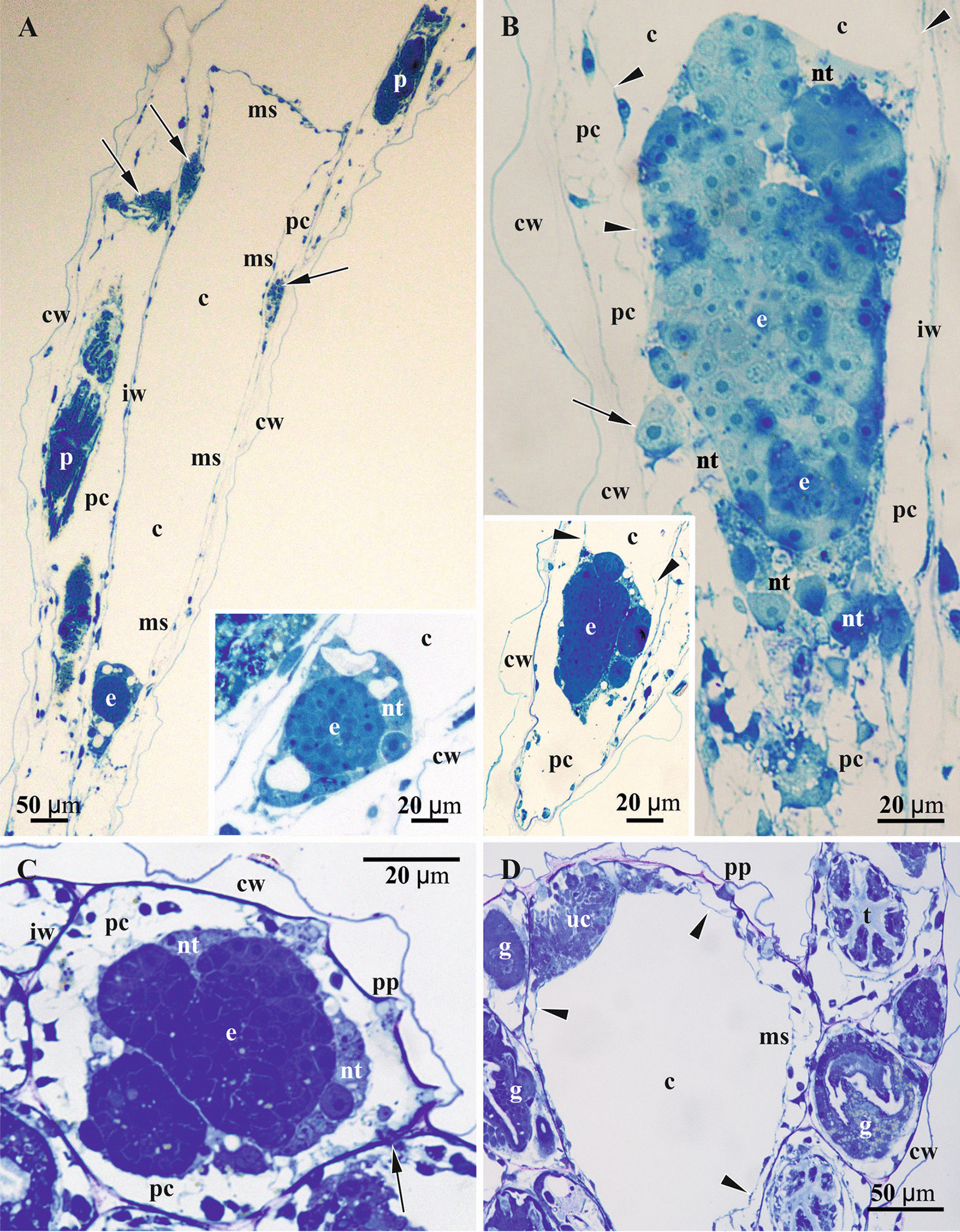
Details of gonozooid anatomy and early embryogenesis in *Crisiella producta* (light microscopy)*.* **a** Longitudinal section of autozooid with functional polypide (to the left), developing autozooid with a polypide bud (upper right corner) and developing gonozooid in between; gonozooid contains extended membranous sac enveloping nearly empty coelomic cavity and early embryo surrounded by nutritive tissue on its bottom (‘upper cell complex’ in pseudocoel shown by arrows; distal, not yet calcified wall of gonozooid is out of image). **a** and insert, Early primary embryo surrounded by developing placental analogue (stage corresponding to Fig. 4e). **b** Gonozooid with late primary embryo and two secondary embryos surrounded by nutritive tissue including several large cells and partly incorporating membranous sac (large pseudocoelomocyte in pseudocoel shown by arrow; gonozooid roof not visible); insert, Gonozooid with primary embryo starting fission (stage corresponding to Figs. 4f and 5c); in both inserts light areas presumably indicate degenerating cells. **c** Cross-section of gonozooid with primary embryo starting fission; membranous sac is non-recognizable, being incorporated into nutritive tissue (plugged communication pore in interzooidal wall shown by arrow). **d** Cross-section of the branch with gonozooid in its upper part showing large, yet empty, incubation (coelomic) cavity and the ‘upper cell complex’ in the pseudocoel. In all zooids the pseudocoel is filled with a network of large and irregular, darkly-stained ‘mesothelial’ cells; in **b**, insert and **d** membranous sac shown by arrowheads. c, coelom; cw, external cystid wall; e, primary embryo; g, gut; iw, interzooidal wall; ms, membranous sac; nt, nutritive tissue; p, polypide; pc, pseudocoel; pp, pseudopore; t, tentacle; uc, ‘upper cell complex’

**Fig. 7:**
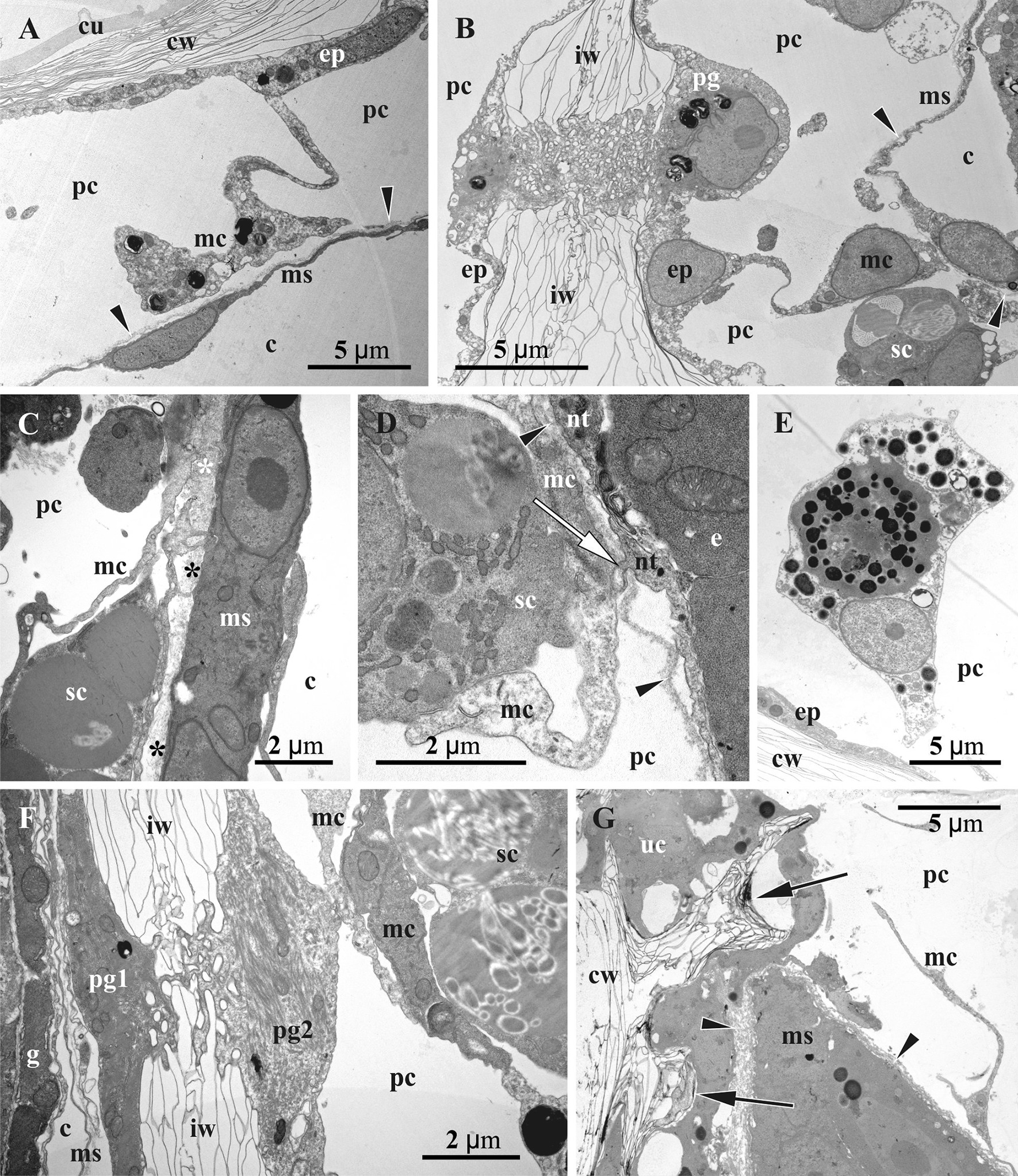
Details of gonozooid ultrastructure in *Crisiella producta* (**a**‒**d**, **f**) and *Crisia eburnea* (**e**, **g**) (TEM). **a** ‘Mesothelial’ cell connecting epithelium of the cystid wall and non-altered membranous sac (here and elsewhere basal lamina of the membranous sac shown by arrowheads). **b** Interzooidal wall (autozooid to the left) with communication pore plugged by a pore-cell, and ‘mesothelial’ cells connected with cystid epithelium and membranous sac. **c** Processes of ‘mesothelial’ cells passing through basal lamina (asterisks) and contacting binucleate cell of membranous sac; **d** ‘Mesothelial’ cell adjacent to basal lamina of former membranous sac (short process of the nutritive tissue piercing it and contacting ‘mesothelial’ cell indicated by white arrow). **e** Presumed pseudocoelomocyte in the pseudocoel. **f** Interzooidal wall between autozooid (to the left) and gonozooid with communication pore plugged by two pore-cells (one of them contacting ‘mesothelial’ cells). **g** Wall of gonozooid with two mural spines (arrows). c, coelom; cu, cuticle; cw, organic matrix of calcified wall; e, early secondary embryo; ep, epidermal lining of cystid walls; g, gut of autozooid; iw, interzooidal wall; mc, ‘mesothelial’ cell; ms, membranous sac; nt, nutritive tissue; pc, pseudocoel; pg (1 & 2), pore-cells plugging communication pore; sc, storage cells; uc, ‘upper cell complex’

Each cyclostome autozooid consists of a tubular cystid—body wall of ectocyst (consisting of external cuticle and calcified wall) underlined by epithelial endocyst, and a polypide comprising a retractile crown of ciliated tentacles (lophophore) and U-shaped gut (Figs. 4, 5a‒c, 6a, 7a). The polypide is suspended within the membranous sac—a free peritoneal wall with basal membrane dividing the zooidal cavity into an enclosed coelom with a polypide, and a pseudocoel—space between the cystid wall and membranous sac (also termed endo- and exosaccal cavities, respectively). The latter acts as a hydrostatic system: contraction of its annular muscles decreases its volume with a corresponding increase in coelomic fluid pressure, resulting in lophophore protrusion via zooidal orifice situated in the centre of terminal membrane in crisiids. Large paired retractor muscles retract the lophophore (Figs. 4a, c, d, 5a, b, 6a).

### Gonozooid

Gonozooids in *Crisia eburnea* and *Crisiella producta*, although differing in shape and size (Figs. 1, 2), do not show detectable differences in anatomy and ultrastructure. In Crisiidae they develop from rather small tubular autozooidal female polymorphs (with a polypide having rudimentary gut) to large club-like incubation chambers. This transformation is accompanied by (1) considerable cystid enlargement (inflation and elongation), and development of a flattened terminal ooeciostome (tube for larval release terminated with ooeciopore—skeletal aperture homologous to autozooidal skeletal aperture) (Figs. 1, 2), (2) irreversible degeneration of the polypide, and (3) expansion and radical cell rearrangement of the membranous sac, which becomes part of the placental analogue (compare Figs. 3, 4, 5, 6). The fate of the zooidal orifice (non-skeletal opening through which the tentacles are protruded) is unknown, but the folded vestibulum persisted although no lumen was detected inside (Fig. 10, insert).

**Fig. 8:**
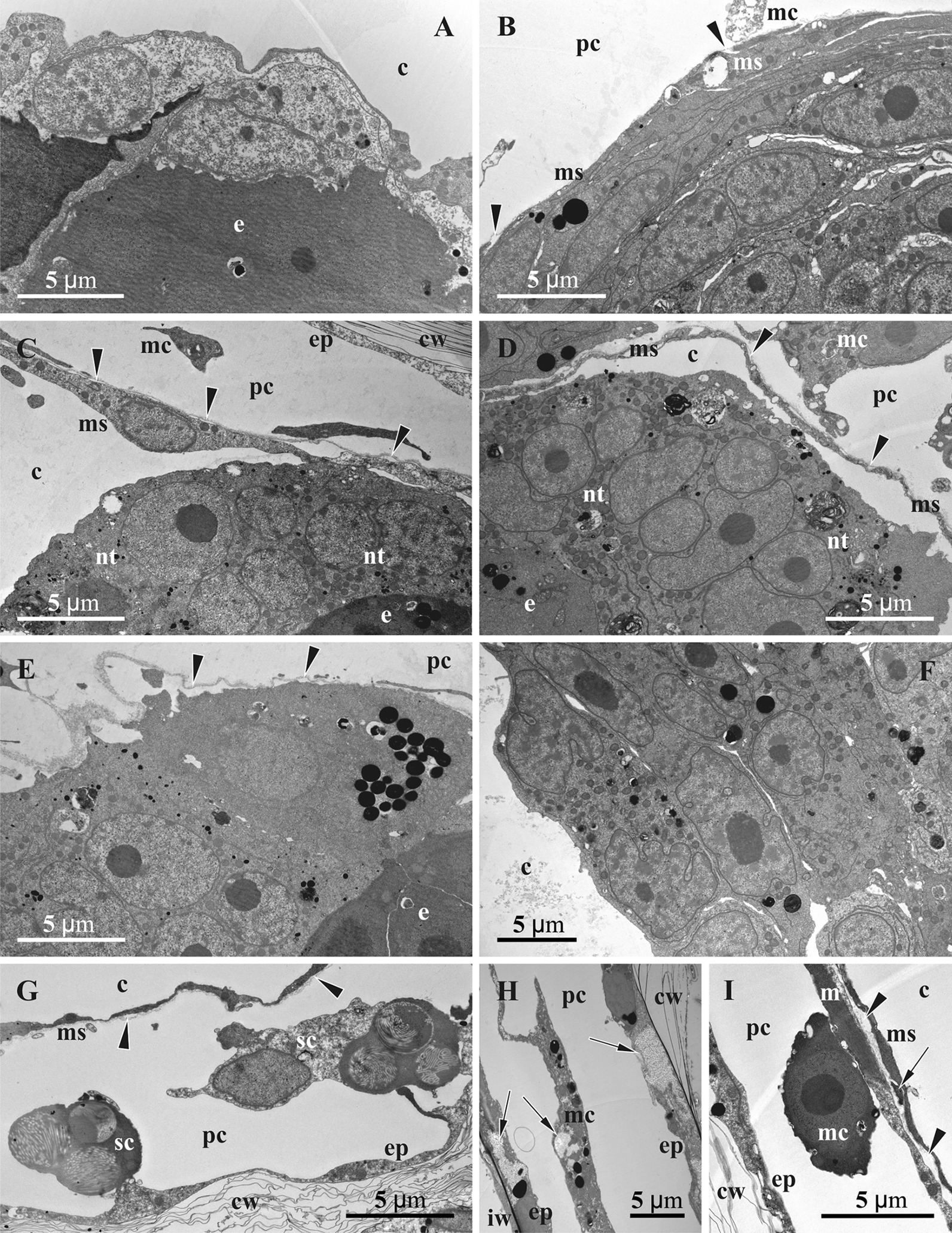
Early developmental stages of the nutritive tissue surrounding early primary embryo, and microanatomical details of *Crisiella producta* (**a**‒**g**, **i**) and *Crisia eburnea* (**h**) (TEM). **a** Early multilayered ‘envelope’ consisting of mononucleated cells with cytoplasm of contrasting electron density (membranous sac is out of view). **b** Fully-formed multilayered ‘envelope’ of mono- and binucleate cells with thin membranous sac lining on its periphery (here and elsewhere basal lamina of the membranous sac shown by arrowheads). **c** Partly shown embryo surrounded by the nutritive tissue contacting wall of non-altered membranous sac. **d** Peripheral part of embryo (left corner) and nutritive tissue surrounded by the free wall of non-altered membranous sac. **e** Embryo lined by multi- and mononucleated cells of modified membranous sac becoming a part of the nutritive tissue. **f** Mono- and multinucleated cells in the roof of the membranous sac. **g** Storage cells in the pseudocoel. **h** ‘Fine-grained’ areas (arrows) in the ‘mesothelial’ and epithelial cells. **i** Non-altered wall of the membranous sac with adjoined longitudinal muscle and ring muscle (arrow) embedded in the basal lamina in young gonozooid (large ‘mesothelial’ cell is visible close to longitudinal muscle). c, coelom; cw, external cystid wall; e, primary embryo; ep, epidermal lining of cystid walls; iw, interzooidal wall; m, longitudinal muscle; mc, ‘mesothelial’ cell; ms, membranous sac; nt, nutritive tissue; pc, pseudocoel; sc, storage cell

Similar to the autozooid, the cavity of the early developing gonozooid is divided into the ‘outer’ pseudocoel and ‘inner’ coelom separated by the wall of the membranous sac (Figs. 3, 4, 5, 6, 7, 8). Multiplication of the embryos via polyembryony and development of the placental analogue (see below) resulted in a strong reduction of both cavities, which became diminished to slit-like lacunae.

In the pseudocoel of both, auto- and gonozooids, an irregular network of presumably mesothelial cells (“mesenchymatous cells” of Borg [44], p. 229) is present, being rather dense in some areas (Figs. 5a‒f, 6). It is comprised of numerous irregular-shaped cells with long processes that contact the cystid wall epithelium and/or the membranous sac as well as each other (Figs. 7a‒d, f, g, 8d, h, i, 11h). Cell size and shape, the electron density of the cytoplasm (mostly electron-translucent) and organelle composition varied in these cells, which contained an elongated nucleus, autophagosomes and a range of inclusions (small vacuoles, lipid droplets, etc.). Some epithelial cells of the body wall also formed long processes contacting the ‘mesothelial’ cells.

In both autozooids and gonozooids some of these ‘mesothelial’ cells are located adjacent to the basal membrane of the membranous sac. In gonozooids they were more numerous, usually flattened and partially covering the membranous sac as an outer lining (Figs. 7a‒d, g, 11g, h). These cells appear to be rather active, possessing a large nucleus mostly filled with euchromatin, numerous mitochondria, often cisternae of rough endoplasmic reticulum (RER) and various inclusions—autophagosomes, lipid droplets (black in TEM images), protein platelets (grey), microvesicles with electron-transparent content, etc. Their cytoplasm varied from almost electron-translucent to electron-dense. Noteworthy, some processes of the ‘mesothelial’ cells passed through the basal lamina of the membranous sac and contacted cells/tissue below it (Fig. 7c). In turn, the latter also formed short processes penetrating the basal lamina and contacting adjacent ‘mesothelial’ cells (Fig. 7d). In contrast, in autozooids the ‘mesothelial’ cells associated with the membranous sac were smaller and contained fewer organelles. No connections with the cells of the membranous sac were detected.

Some large ‘mesothelial’ cells contained few to many voluminous (2‒3 µm in diameter) spherical inclusions, either homogeneous (Fig. 7c) or with characteristic ‘striated’ appearance (presumably protein platelets with paracrystalline structure) (Figs. 7b, f, 8g). Such cells (termed as ‘storage cells’ here and elsewhere) can be found in any part of the pseudocoel and were more numerous in gonozooids. Their cytoplasm was usually electron-dense, with a large active nucleus, numerous free ribosomes, mitochondria and RER cisternae. Similar ‘striated’ inclusions were sometimes visible in the epithelial cells of the cystid wall and were often detected in the cells of early larvae (Fig. 10d). At the same time, they were never observed in the cells of the membranous sac (of both autozooids and gonozooids) or of the polypide. In addition, some epithelial cells of the body wall and some ‘mesothelial’ cells contained large ‘fine-grained’ electron-translucent zones (Fig. 8h) in both auto- and gonozooids. Similar zones were also detected in some larval cells (Fig. 10f).

Large solitary cells of irregular shape (that could be termed ‘pseudocoelomocytes’) were occasionally observed in the pseudocoel of both auto- and gonozooids (Figs. 6b, 7e). Their cytoplasm varied in electron density, containing large spherical or lobate nucleus and organelles (RER cisternae, mitochondria, vacuoles, lipid droplets and other inclusions) that were distinctly more numerous than in the ‘mesothelial’ cells.

In autozooids and developing gonozooids of both studied species, large, darkly stained (in histological sections) round, oval or cubic cells (termed together the ‘upper cell complex’ here and elsewhere) were detected in the pseudocoel surrounding the atrial sphincter and the roof of the membranous sac (including attachment organ) (Figs. 4a‒c, 5a, 6a, d). These cells either form a prominent incomplete ‘ring’-mass or few smaller groups, exhibiting an electron-dense cytoplasm, large nucleus, mitochondria, numerous free ribosomes and RER cisternae. A few of them also bear lipid droplets, autophagosomes and other inclusions, sometimes with a homogeneous or ‘striated’ content (Fig. 10g). In the upper part of the developing gonozooids, the ‘upper cell complex’ filled the pseudocoel above the roof of the membranous sac with thickened basal lamina (Figs. 6a, d, 10g, h). Cells of the membranous sac exhibited a similar ultrastructure to the cells of the ‘upper cell complex’ above them. Later they become hypertrophied and multiplied their nuclei. The basal lamina in the roof of the membranous sac was thicker, more electron-dense and deeply folded than elsewhere (Fig. 10h). Both cells of the membranous sac and the ‘upper cell complex’ bore processes, some of which pierced the basal membrane and contacted the cells on the opposite side (Fig. 7c).

Communication pores are represented by a canal in the interzooidal wall with a three-dimensional lattice/labyrinth of small calcified bars (spines). This canal is also filled by 1‒2, presumably specialized epidermal cell(s) (Figs. 4d, 6c, 7b, f). In comparison with the ordinary epithelial cells, the pore-cells are larger, contain a large lobate nucleus and, often, microfilaments (sometimes numerous). The cytoplasm’s electron density strongly varied from transparent to very dense. Some pore-cells possessed numerous mitochondria, autophagosomes and several Golgi complexes together with small peripheral vacuoles. If a single cell filled the communication pore, its opposite terminal parts were wider than the central narrow part (filling the ‘labyrinth’ canal), thus sometimes giving the pore-cell a ‘dumbbell’ shape (Fig. 7b). When two cells plugged the communication pore, their cytoplasms may drastically differ in electron density (Fig. 7f). Some pore-cells were in contact with processes of the ‘mesothelial’ cells.

Mural spines (internal outgrowths of the skeletal wall directed into the zooidal cavity) are often developed in the gonozooid roof (Figs. 7g, 11i). They vary in size and have complex (hooked, forked or branched) shape, being surrounded by the cells of the ‘upper cell complex’. No muscles were detected in association with the mural spines in the studied material.

Very thin annular (ring) muscles are embedded in the basal lamina of the membranous sac in developing gonozooids. Longitudinal muscles were also found close to the membranous sac in the pseudocoel (Fig. 8i), sometimes adjoining its basal lamina. No muscular elements were present in mature gonozooids.

### Oogenesis

Ovaries were found in *Crisiella producta* only. The earliest oocytes (20 × 16 µm in diameter) were detected in young gonozooids (= autozooidal polymorphs) with the polypides having eight (as in autozooids) short tentacles and an underdeveloped gut with triradial pharynx. No food was present inside the gut. In our material the early gonozooids always had only one ovary associated with the basal part of the gut and containing a single oocyte surrounded by a single layer of flattened and cubic follicular cells. Mature oocytes (25 × 20 µm) are oligolecithal with few yolk granules in the cytoplasm, and have a large nucleus with prominent nucleolus (Fig. 5a). Incidentally, single oocytes (20 μm in diameter) were found inside the membranous sac, unconnected to the polypide. Sperm was not detected in the material studied.

### Early embryogenesis and formation of nutritive tissue

We found early primary embryos in *Crisiella producta*. They were located in the proximal part of gonozooid at the bottom of the membranous sac (Figs. 5b‒d, 6a, inserts). Early embryogenesis was accompanied by polypide degeneration, but no residual polypide (‘brown body”) was detected except for some resorbing retractor muscles close to the developing embryo. In contrast, the membranous sac was greatly expanded, enveloping the coelomic cavity that occupied most of the growing gonozooid volume. The ‘upper cell complex’ was present inside the still voluminous pseudocoel around the upper part of the membranous sac (Fig. 6a, d).

We did not observe the initial stages of cleavage. The early primary embryo found (diameter approximately 30 µm) consisted of about ten large round blastomeres with loose arrangement (Fig. 5b). The blastomeres possessed an electron-dense cytoplasm and large round nucleus together with multiple free ribosomes, mitochondria and RER cisternae (partly visible in Fig. 8a, c, e). Some blastomeres were clearly resorbing.

The early primary embryo is encircled by a massive, multilayered ‘envelope’ of large flattened cells that, in turn, are surrounded by the non-modified wall of the membranous sac (Figs. 3a, 5b, c, 8a, b). These ‘enveloping’ cells were rather variable in size, thickness and electron density of their cytoplasm and the number of organelles. Many of them contained numerous mitochondria; some had autophagosomes. Their nuclei were large, round or lobate with large nucleoli. Some of these cells were binucleate. The wall of the membranous sac was either free or adjoined the multilayered ‘envelope’ surface (Figs. 3a, 8b‒d).

At a later stage, coenocytes (sensu [75]) form via nuclear multiplication and cytoplasmic growth on the periphery as well as inside the multilayered cellular ‘envelope’ (Fig. 5d). The number, size, shape and position of the nuclei indicate a coenocytic, not syncytial, mechanism of formation (Fig. 8c‒f). Similar changes affected some cells of the membranous sac wall. They enlarged, became bi- or multinucleated and were incorporated into the multilayered structure around the embryo (Figs. 3b, 8e). The embryo itself becomes a solid round morula (about 50 µm in diameter) of large, round or cubic cells (Fig. 5e).

As the primary embryo grows, the multilayered ‘envelope’ consisting of mostly individual cells and some coenocytes is gradually transformed into “nutritive tissue” (term of Harmer [42], p. 133) (Figs. 3b, C, 5e, 8c, d). The individual cells are still recognizable because of their dark-stained/electron-dense cytoplasm. These cells become resorbed later on, leaving large ‘empty spaces’ that are visible in histological sections (Fig. 6a, inserts, c). The wall of the membranous sac surrounding the nutritive tissue is still partly free, partly incorporated into the nascent placental analogue (Figs. 5e, f, 6a‒c, inserts, 8c, e). The upper (distal) area of the membranous sac wall that enveloped ‘empty’ coelomic space retains its cellular structure. The entire membranous sac is surrounded by the basal lamina (Fig. 3c).

Eventually, the proximalmost part of the membranous sac becomes entirely incorporated into the newly established nutritive tissue without detectable cell membranes inside (Fig. 3c). This tissue is rather uniform ultrastructurally, with electron-translucent cytoplasm and numerous mitochondria, free ribosomes, Golgi complexes and RER cisternae, indicating prominent synthetic activity (Fig. 8c, d). Vacuoles with various content, autophagosomes and numerous lipid droplets of various sizes are spread through the cytoplasm. The numerous large nuclei, round as well as lobate, were mostly filled with euchromatin. Small lipid droplets were also recorded in some peripheral blastomeres of the embryo embedded in the nutritive tissue (Fig. 8c, d).

As embryos multiplied via fission of primary embryo (see below), the cells in the roof of the membranous sac became multinuclear with large lobate nuclei, further acquiring coenocytic structure (Fig. 8f) (see also above). Similar changes occurred in the ‘upper cell complex’. Nonetheless, non-altered peritoneal cells make up most of the membranous sac wall between its roof and the lowest part incorporated within the nutritive tissue around the primary embryo (Fig. 3d).

### Embryonic fission

The establishment of the nutritive tissue is accompanied by embryonic growth and initial embryonic fission. Formation, growth and development of the numerous secondary embryos that constrict off the primary embryo (polyembryony) occur inside the growing nutritive structure, which functions as a placental analogue (Fig. 3d, e).

When starting fission, the late primary embryo has an irregular shape forming deep slits separating rounded and oval prospective secondary embryos (Figs. 3d, 5f, 6b, insert, c). Similar to the early primary embryo, neither cellular layers nor definite zone(s) of cell proliferation were detected in this growing embryonic ‘mass’ (Figs. 5f, 6b, insert, c). Newly formed secondary embryos differ in shape and size, and usually present with young larvae in the same mature gonozooid (Figs. 2b, c, 3e, 6b, 10a, b).

### Secondary embryos and early larvae

**Fig. 9:**
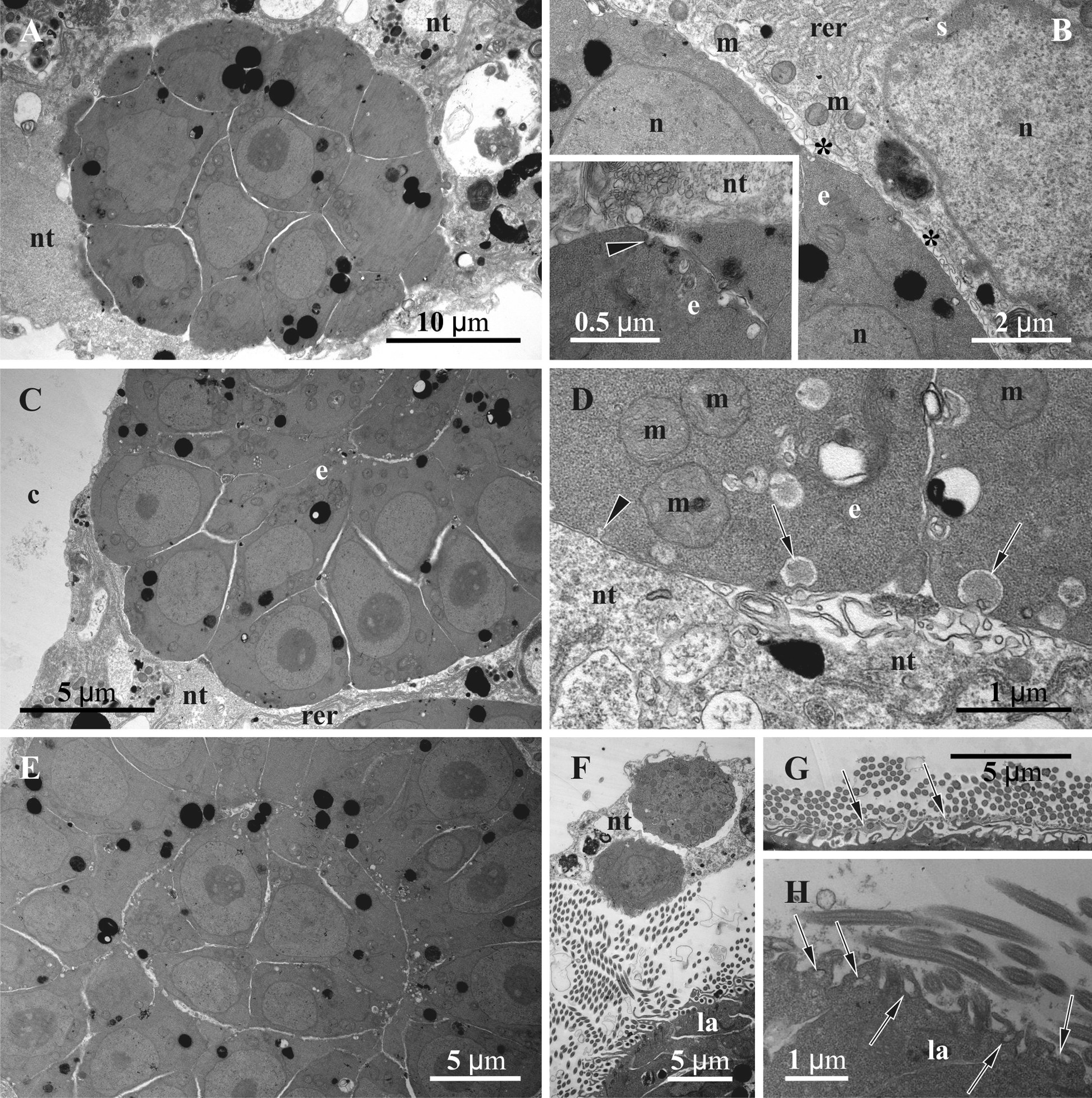
Details of ultrastructure of the secondary embryos and early larvae in *Crisiella producta* (**a**‒**e**, insert) and *Crisia eburnea* (**f**‒**h**) (TEM). **a** Cross-section of the early secondary embryo surrounded by nutritive tissue. **b** Peripheral part of the early secondary embryo surrounded by nutritive tissue with folded membrane (asterisks); insert, plasma membrane of embryonic cells with forming coated pit (arrowhead) and microvesicles. **c** Part of the early secondary embryo starting delamination. **d** Apical membrane of peripheral embryonic cells showing forming coated pit (arrowhead) and (supposedly) endocytic microvesicles (arrows); adjacent membrane of the nutritive tissue strongly folded. **e** Partial view of the secondary embryo showing central and peripheral zones during delamination (numerous electron-translucent microvesicles are especially abundant around the ‘inner’ cells). **f** Periphery of the early ciliated larva surrounded by nutritive tissue with two large round solitary cells (microvilli around basal parts of cilia are clearly visible). **g** Surface of early larva with glycocalyx (arrows) above microvilli. **h** Surface of early larva with numerous microvilli, ‘endocytotic’ pits and microvesicles (arrows) between cilia bases. Electron-dense lipid droplets are visible in embryonic and larval cells as well as in the nutritive tissue. c, coelom; e, secondary embryo; la, larva; m, mitochondrion; n, nucleus; nt, nutritive tissue; rer, rough endoplasmatic reticulum

Young secondary embryos were round or oval solid morulae of various sizes (smallest embryos were 25 × 20 µm and 30 × 20 in *Crisia eburnea* and 30 µm and 35 × 25 µm in *Crisiella producta*) showing no distinct cell layers (Figs. 6b, insert, c, 9a, 10a, b). Their blastomeres with large nuclei were similar in shape and size to the blastomeres of the primary embryo. Some showed signs of endocytosis: coated pits and small vesicles with various content were associated with the plasma membrane (Fig. 9b, insert, d). Interestingly, these pits and vesicles were present in both peripheral and internal blastomeres. Possible signs of exocytosis (as tiny foldings of plasma membrane) were incidentally detected on the surface of the nutritive tissue (Fig. 9b, d).

**Fig. 10:**
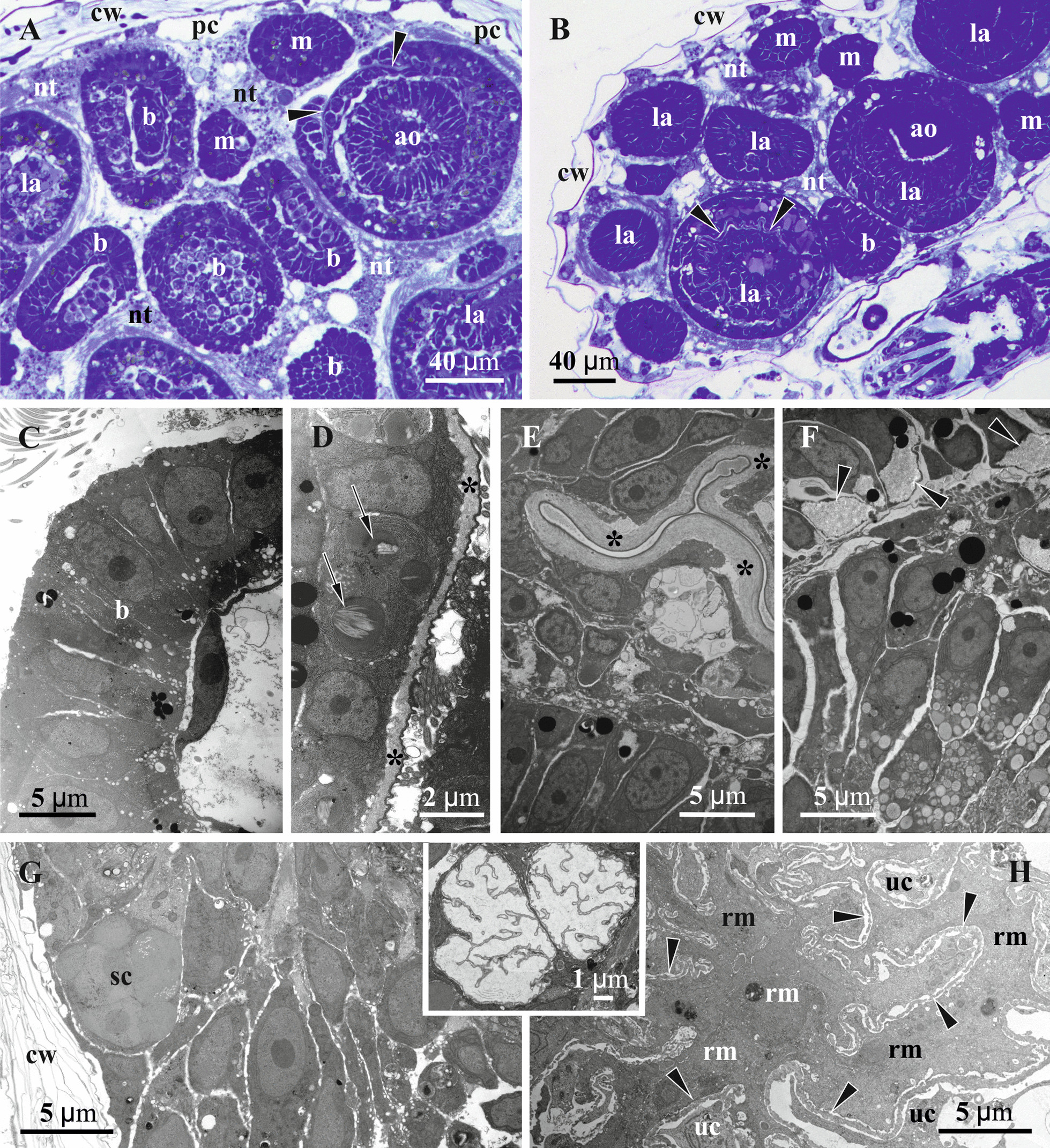
Details of larval and gonozooid structure in *Crisia eburnea* (**a**, **h**) and *Crisiella producta* (**b**‒**g**) (**a**, **b**, light microscopy, **c**‒**h**, TEM). **a**, **b** Oblique section of gonozooid showing secondary embryos and early larvae embedded into the nutritive tissue (folded cuticular area of the larval apical pole shown by arrowheads). **c** Part of bilayered secondary embryo with central cavity, peripheral columnar and flattened ‘inner’ cell lining. **d** Apical part of early larva with cuticular ‘cap’ (asterisks) (arrows show granules with ‘striated’ content). **e** Part of early larva with folded cuticular ‘cap’ (asterisks). **f** Elongated cells of the larval adhesive sac with numerous round inclusions and more peripheral cells with ‘fine-grained’ content (arrowheads). Electron-dense lipid droplets are visible in embryonic and larval cells. **g** ‘Upper cell complex’ filling space between gonozooid wall (lower left corner) and the roof of membranous sac (not shown); folded cuticular wall of remained vestibulum shown in insert. **h** Cross-section of the uppermost part of folded roof of coenocytic membranous sac surrounded by thick basal lamina (arrowheads) and ‘upper cell complex’. ao, adhesive organ of larva; b, bilayered secondary embryo; cw, external cystid wall; la, early larva; m, morula-like secondary embryo; nt, nutritive tissue; pc, pseudocoel; rm, roof of membranous sac; sc, storage cells; uc, ‘upper cell complex’

**Fig. 11:**
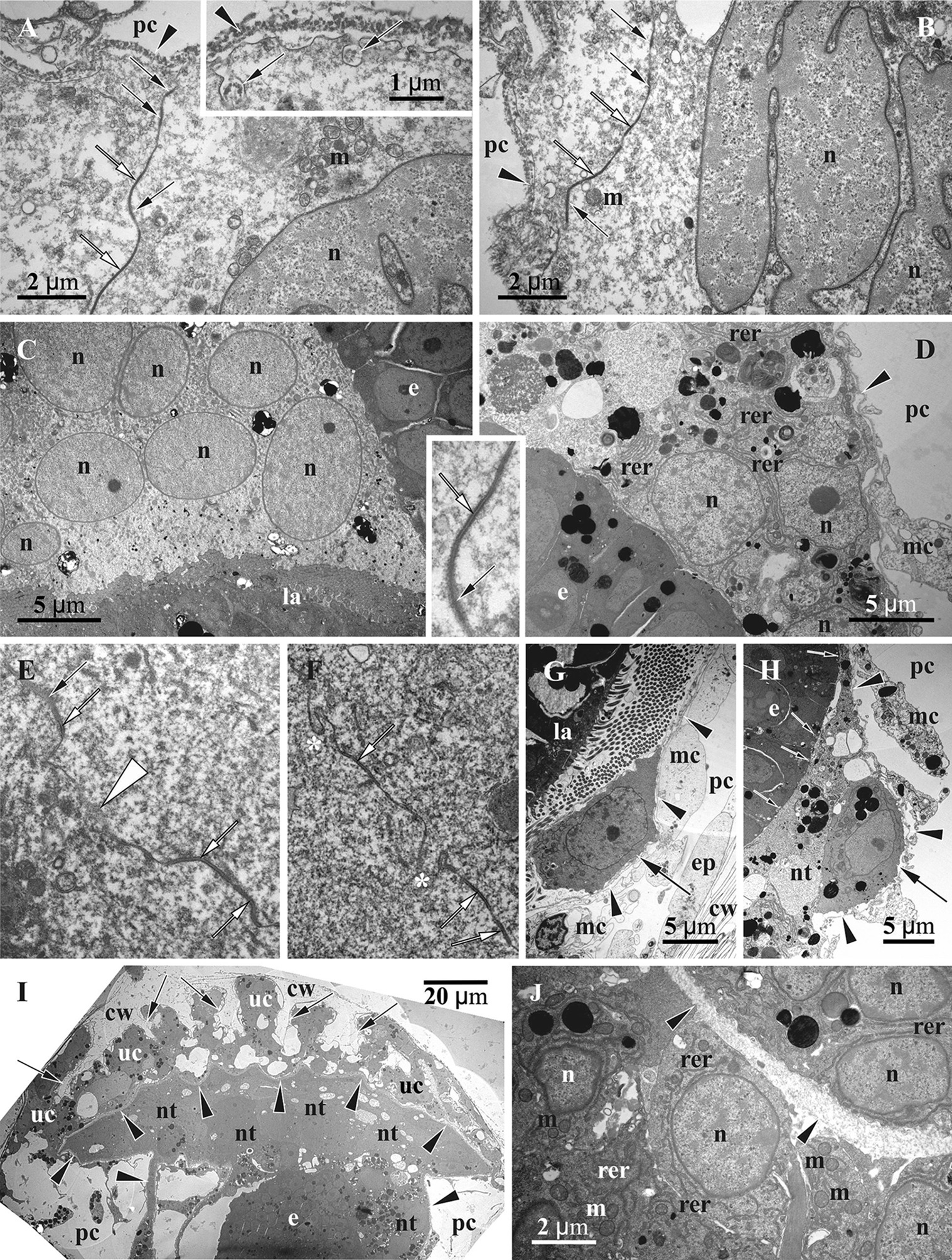
Details of ultrastructure of fully-formed nutritive tissue in *Crisia eburnea* (**a**‒**c**, **e**‒**g**, **i**, inserts) and *Crisiella producta* (**d**, **h**, **j**) (TEM). **a**, **b**, Contact of two coenocytic ‘elements’ with lobated nuclei and electron-translucent cytoplasm mostly free of organelles and tight (black arrows) and adherens (white arrows) junctions connecting neighbouring plasma membranes; insert, presumed endocytotic vesicles (arrows) in the plasma membrane facing the pseudocoel (here and elsewhere the basal lamina of the former membranous sac shown by arrowheads). **c** Part of the nutritive tissue with electron-dense cytoplasm and multiple nuclei in between larvae and secondary embryo. **d** Periphery of the embryo and adjacent part of the nutritive tissue with electron-dense cytoplasm, two nuclei, multiple autophagosomes and other inclusions and RER; insert, tight (black arrow) and adherens (white arrow) junctions connecting neighboring plasma membranes. **e** Cytoplasmic bridge (white arrowhead) between coenocytic ‘elements’ (tight and adherens junctions indicated by black and white arrows, respectively). **f** Interdigitations (asterisks) between coenocytic ‘elements’ (adherens junctions indicated by white arrows) (in insert and **e** and **f**, the adherens junctions are 50 nm wide). **g**, **h**, Solitary cells (arrows) with electron-dense cytoplasm incorporated into the nutritive tissue (basal lamina of the membranous sac shown by arrowheads; presumed endocytotic vesicles in embryonic cells shown by small arrows). **i** Longitudinal section of the terminal part of gonozooid with folded roof of modified (coenocytic) membranous sac covered by the ‘upper cell complex’ (also with coenocytic structure) (skeletal spines shown by arrows); secondary embryo is recognizable in lower part of image. **j** Coenocytic ‘upper cell complex’ (to the left) separated by basal lamina from coenocytic membranous sac. e, secondary embryo; ep, epithelial cell; cw, cystid wall; la, larva; m, mitochondrion; mc, ‘mesothelial’ cell; n, nucleus; nt, nutritive tissue; rer, rough endoplasmatic reticulum; pc, pseudocoel; uc, ‘upper cell complex’

Growth and development of the morulae were further accompanied by delamination via establishment of the ‘inner’ and peripheral cell zones first (Fig. 9c, e). While embryonic cells in both zones are similar, numerous small, electron-translucent vacuoles appear in association with plasma membranes, marking a ‘border’ between ‘inner’ and peripheral embryonic cells facing each other (Fig. 9e). The function of these vacuoles is unclear. Moreover, the apical membrane of the peripheral embryonic cells exhibits small vesicles and pits, and the abutted membrane of the nutritive tissue is folded in some areas indicating both endo- and exocytosis, correspondingly (Figs. 9b, d, 11h).

At the next stage, secondary embryos became elliptical, consisting of 1‒2 layers of larger peripheral, mostly columnar and prismatic cells lined by the smaller and flattened, internal cells (former ‘inner’ cells) (Fig. 10a, c). In addition, cells of ‘intermediate shapes’ (cubic or oval) sometimes occur in between the peripheral and ‘inner’ cells. Flattening of the latter resulted in the formation of the central slit-like cavity, which subsequently became more voluminous and moved to the future ‘animal’ embryo pole because of the active cell divisions on the opposite pole. No signs of cell degradation were recorded in the central part of the embryo. Both peripheral and ‘inner’ cells have electron-dense cytoplasm containing large mitochondria. Throughout the above stages, the embryos are embedded in the nutritive tissue.

Next, two-layered embryos form a deep invagination (future adhesive organ) at the ‘vegetal’ pole, reducing the central cavity (Fig. 10a, b), and begin to form a ciliary corona with cells containing numerous mitochondria and common Golgi complexes (Figs. 9f, 11c, g). The ciliated cells develop numerous long (up to 5 µm) microvilli near their bases, which are often branched and anastomosed (Fig. 9f‒h). Abundant pits and microvesicles together with some pinocytotic channels were visible in this zone, indicating active endocytosis between the ciliary bases (Fig. 9h). Ciliated areas often bore a flocculent glycocalyx of varying thickness located around the cilia bases above the microvilli (Fig. 9g). Some non-ciliated peripheral larval cells develop short, irregular microvilli-like projections as well.

At the same time the thick cuticular cover develops at the larval ‘apical’ pole (Fig. 10a, b, d). The cuticle consists of an electron-translucent, finely-fibrous, thick inner layer and electron-dense, very thin outer layer (Fig. 10d, e). The long thin processes of the larval cells occasionally penetrate the inner cuticle layer. Finally, the deep circular folding of the cuticular ‘cap’ results in the formation of an apical invagination (Figs. 10a, e).

Cells of the early larvae have large spherical nuclei with a prominent nucleolus and mostly filled with euchromatin. They have electron-dense cytoplasm, often with numerous RER cisternae, free ribosomes and mitochondria. Many cells also contained specific ‘fine-grained’ areas in their cytoplasm (Fig. 10f) similar to those recorded in some epithelial and ‘mesothelial’ cells (Fig. 8h). Lipid droplets occur throughout the cytoplasm together with large protein granules having a ‘striated’ paracrystalline appearance (Fig. 10d) comparable (although smaller) to those found in some ‘storage’ cells (Figs. 7b, f, 8g). Cells of the forming adhesive organ contained numerous spherical granules (presumably proteinaceous), homogeneous or ‘striated’, and smaller oval granules with ‘opalescent’ content (Fig. 10f). The maximum recorded size of developing larvae was 115 × 110 µm and 120 × 100 µm in *C. eburnea* and 100 × 75 µm in *C. producta*.

### Fully-formed placental analogue

Embryonic multiplication and growth were accompanied by a corresponding distal expansion of the nutritive tissue, gradually incorporating the rest of the membranous sac wall, which still retains its basal lamina. The nutritive tissue with embedded embryos and larvae finally occupies most of the gonozooid internal space, leaving only a slit-like pseudocoel between the basal lamina of the membranous sac and the cystid wall (Figs. 3e, 10a, b, 11i). The fully-formed placental analogue comprised large coenocytic ‘parts’/‘areas’ separated by a plasma membrane and interconnected by cytoplasmic bridges (Figs. 3e, 11a, b, e, f). Large solitary cells are also incorporated in the analogue (Figs. 3e, 11g, h). These cells are usually positioned at the periphery close to the basal membrane and are round or oval with a slightly convoluted membrane. They have a distinctly electron-dense cytoplasm and large nucleus (sometimes two), numerous free ribosomes, mitochondria and RER cisternae. Some also contained lipid droplets and/or protein platelets.

The ultrastructure of the coenocytic ‘parts’ differs. All possess large, round or lobate nuclei (sometimes in groups) filled either with euchromatin or with unevenly distributed heterochromatin (Fig. 11a‒d). Some coenocytes have an electron-translucent cytoplasm containing few Golgi complexes and RER cisternae, rare lipid droplets and protein platelets and scattered vacuoles (Fig. 11a, b). Scattered large multivesicular-like bodies and autophagosomes are present, whereas free ribosomes and mitochondria are not very abundant. Incidentally, signs of presumed endocytosis were recorded at the coenocyte periphery below the basal lamina (Fig. 11a, insert).

In contrast, other coenocytic areas appear to be much more ‘active’, having a more electron-dense cytoplasm with numerous free ribosomes, mitochondria and various inclusions, Golgi complexes and distinctly more developed RER (Fig. 11c, d). Lipid droplets and protein platelets (when present) are usually larger too, as are the autophagosomes. Some areas also contain irregular-shaped RER cisternae of various sizes filled with electron-dense material, and vacuoles with flocculent material. Areas with an ‘intermediate’ appearance are also present. No clear distributional pattern of the coenocytic ‘parts’ with contrasting ultrastructures was evident through the gonozooid.

Neighbouring coenocytic areas often ‘overlap’ or form multiple, sometimes rather complex infoldings (interdigitations) (Fig. 11f). In both instances, we recorded tight and adherens junctions (often alternating in a row) (Fig. 11a, b, insert, e, f). Both interdigitations and junctions tend to be more frequent in the terminal (distal) part of the gonozooid (see also below). The thickness of the placental analogue varies regardless of its position. Narrow areas of the nutritive tissue are present either on its periphery or between neighbouring embryos/larvae, preventing them from contacting each other (Figs. 3e, 9c) (minimum thickness about 200 nm). Thickened parts are widespread and sometimes composed by two (or more) adjacent coenocytes, often with contrasting ultrastructure. In both cases, coenocytes formed short protuberances piercing the basal lamina and contacting the ‘mesothelial’ cells lining it (Fig. 7d).

In the fully-formed functioning gonozooid the ‘upper cell complex’ and the cells of the roof of the membranous sac together form a thick coenocytic ‘cap’, separated by a remarkably thick and folded basal lamina into upper and lower zones (Figs. 3e, 7g, 10h, 11i). Each zone comprises several coenocytic ‘parts’ having an electron-dense, ribosome-filled cytoplasm with abundant mitochondria and RER elements, and numerous round or lobate (presumably, dividing) nuclei (Fig. 11j). They also contain lipid droplets, autophagosomes and, sometimes, granules with ‘striated’ contents (in the ‘upper cell complex’). Inclusions (vacuoles, vesicles, etc.) are generally less numerous in the ‘cap’ compared to the other parts of the placental analogue. The adjacent coenocytic ‘parts’ are tightly packed, showing complex interdigitations with adherens junctions. The remnants of the folded vestibulum are often present here in both young and mature gonozooids (Fig. 10, insert).

## Discussion

### Historical background

Although Borg [44] gave detailed analysis of the previous studies of the cyclostome reproduction, we feel that the landmark works should be mentioned here to facilitate a discussion of our major findings. Smitt [56, 57] was the first to describe and illustrate multiple embryos [as eggs] and larvae [as ciliary embryos] inside broken gonozooids of *Crisia eburnea* and *Tubulipora liliacea* (as *T. serpens*). He termed the soft ‘tissue’ enveloping the embryos as a “mantle”, measured growing embryos and larvae at different stages of their development, and described the formation of the adhesive organ in the latter. He also mentioned that “eggs” develop from the “fatty mass” inside the gonozooid, which he reported as originating in the same way as autozooids. Hincks ([76], p. 418) followed Smitt, stating that gonozooids are enlarged autozooids “modified for … reproductive functions”.

Harmer applied then the new histological technique and was the first to describe cyclostome breeding seasons and sexual reproduction in detail in the six species of three families, Crisiidae [40, 45, 46], Lichenoporidae [41, 47] and Tubuliporidae [42, 48]. He followed gonozooid development in these taxa, detected early germ cells (both male and female) in the early forming zooids, and reported oocytes associated with a rudimentary polypide in young gonozooids. He also described the cleavage and development of the “primary embryo”, whose further growth was accompanied by the formation of the “finger-shaped processes” and “embryonic fission” resulting in multiple “secondary embryos” ([40], p. 200). He also briefly described larval anatomy. Harmer wrote that embryos and developing larvae were embedded in “nucleated protoplasmic reticulum”—the result of the transformation of the multinucleated “follicle” (which he thought was formed from the polypide gut). Considering the massive embryonic multiplication and growth, he correctly suggested a nutritive function of the “protoplasmic network”, comparing it to the placenta of mammals and salps. Harmer [40] was also the first to describe, measure and illustrate cyclostome sperm as well as spermatogonia and spermatocytes. Noteworthy, in explaining the life-history of *Crisia eburnea*, he mentioned that in spring (e.g., in April) sperm was found in the well-developed colonies without gonozooids that (“are proably in most cases of the male sex”), whereas he never found sperm in the colonies with gonozooids [46, p. 145]. Similar statements on “male” and “female” colonies were later published by Robertson [43].

Robertson [43] ‘repeated’ the work of Harmer and examined four crisiid species. This yielded new information on cyclostome reproduction, e.g. she described and illustrated spermatogenic tissue (as testis) as well as a multilayered cellular ‘envelope’ around the primary embryo and detected the formation of the tertiary embryos in one species (a brief review on cyclostome reproduction was also included in [77]).

Calvet [62] included data on sexual reproduction and development of four species from four families (Crisiidae, Tubuliporidae, Lichenoporidae, Oncousoeciidae) in his comprehensive monograph on bryozoan anatomy. The main focus was on cyclostome embryonic development (especially formation of the secondary embryos) and larval structure and their comparison with those in gymnolaemates.

Waters [49–51] also provided some data on cyclostome reproduction based on histological sections of five species from the families Plagioeciidae, Horneridae and Crisinidae, confirming the presence of polyembryony in them.

Some preliminary data on sexual reproduction in Cyclostomata were included in the early work of Borg [52], who also coined the term “gonozoid”, but his ultimate study on cyclostome reproductive anatomy was published in 1926 [44]. Borg described the history of the studies on the incubation chambers in Cyclostomata, and gave the most detailed and precise description of cyclostome sexual reproduction up until that time. Using histological sections, he described its major aspects in 16 species from seven cyclostome families (Crisiidae, Tubuliporidae, Plagioeciidae, Lichenoporidae, Frondiporidae, Fasciuculiporidae, Horneridae) and made a careful comparative analysis, also exhaustively collating his own results with those of earlier authors. The most detailed descriptions were devoted to *Crisia eburnea* and *Crisiella producta*, the two species that we studied here.

Finally, a monograph on cyclostome incubation chambers was published by Schäfer [31]. Although it included some anatomical data on sexual reproduction, including spermato- and embryogenesis as well as larval structure, this work mostly focused on the morphological diversity and evolution of skeletal characters.

### Gonozooid development and structure

#### Polypide and coenocytic ‘cap’

Gonozooid development in cyclostomes is accompanied by a considerable enlargement of the autozooid or (in crisiids) autozooidal polymorph related to the establishment of the ovary (see below). It includes changes in cystid size and shape, disintegration of the polypide and associated organs (retractor muscles and funiculus), and modification of the hydrostatic apparatus and disintegration of its muscular elements. The free distalmost part of the autozooidal cystid (peristome) transforms into an ooeciostome (specialized tube for larval release) with corresponding changes to the vestibulum [40, 42], whose folded cuticle is retained ([62], Pl. 10, Fig. 15, oval ‘rugose bodies’ without designation; our data).

Our study showed that the autozooidal polymorph (presumptive gonozooid) in Crisiidae contains a non-feeding polypide still capable of protrusion. Based on the tentacle length and presence of the vestibulum, an atrial sphincter (although less developed), ligaments (= attachment organ), a tentacle sheath and membranous sac, Borg ([44], p. 418) wrote that in *Crisiella producta* the “fertile polypides … would be very nearly full-grown” (presumably, comparing their size with that in autozooids), although their intestine remains underdeveloped, which he explained by the influence of the female gonad. Indeed, all such polypides in *C. producta* we studied had shortened tentacles (not seen in *C. eburnea*). Nonetheless, whether other crisiids possess a rudimentary or non-altered tentacle crown in young gonozooids should be checked.

The presence of annular muscles in the membranous sac, longitudinal muscles in the pseudocoel (Fig. 8i) and retractor muscles ([44]; our data) proves the ability of the polypide of the prospective gonozooid to expand and retract. This indicates that sperm uptake from the water column is its main function. The same is assumed, for instance, for the cheilostome *Celleporella hyalina*, which exhibits non-feeding dwarf female zooids with a rudimentary polypide and developed hydrostatic apparatus [25, 78]. In both cases the sperm itself should enter the coelom via a supraneural coelomopore (reviewed in [79]). Although Borg [44] described and illustrated a so-called “fertile brown body” in the young gonozooid of *Crisiella producta*, we were unable to confirm its existence, leaving this question open for further investigation. It is possible that the polypide is totally utilized for the needs of the growing nutritive tissue and primary embryo that starts to develop about that time.

In the gonozooid the wall of the membranous sac becomes part of the nutritive tissue enveloping embryos and larvae, while its basal lamina persists. Interestingly, Borg [44] stated that the membranous sac expands to almost the entire gonozooid “enclosing the whole mass of embryos, larvae and nutritive tissue”, and its wall keeps the “ordinary structure” (pp. 425–426). In contrast, Harmer ([40], p. 216) wrote that the membranous sac (as “tentacle sheath”) “probably fuses” with the developing nutritive tissue, and our data are in agreement with this view.

Harmer ([40], p. 21) also was the first to describe and illustrate so-called “distal thickening of the tentacle sheath”, and it was Borg [44], who recognized it as originating from the distal part of membranous sac wall and serving as one of the sources of the nutritive tissue via cell division, transformation and migration. Our data complement this view. The ‘distal thickening’ of Harmer and Borg is what we term the coenocytic ‘cap’ consisting of the strongly modified ‘upper cell complex’ and the thickened roof of the membranous sac separated by the basal lamina. During ‘cap’ formation the cells of these two parts obtain numerous nuclei (also detected by Harmer [40]) and enlarge, thus becoming coenocytes that contact each other via short cytoplasmic processes piercing the basal lamina (Fig. 7c, d). It seems likely that the “multinucleated masses of protoplasm” and at least some of the bi- or multinucleated “giant-cells” described and illustrated by Harmer ([40], pp. 220, 240) as “derived from the thickened distal end of the tentacle-sheath [membranous sac]” are coenocytes which originated from the cells of the membranous sac roof. Borg ([44], p. 343) also saw these cells.

The mural spines of various shapes that we detected as being embedded in the ‘upper cell complex’ in the terminal part of gonozooids (Figs. 7g, 11i), were earlier described in autozooids of crisiids by Weedon and Taylor [80]. Because the attachment organ of the autozooidal polymorph disappears during transformation to the gonozooid, their probable function is to anchor the coenocytic ‘cap’ when it becomes part of the massive nutritive tissue enveloping multiple embryos and larvae.

The passage through which mature larvae escape from the gonozooid was not recognizable in our sections, and we doubt that such a permanent passage exists. Potentially, mature larvae with actively functioning ciliature could rupture the coenocytic ‘cap’, reaching the ooeciostome. Moreover, we were unable to confirm the existence of a so-called “larval chamber” isolating ready-to-leave larvae in the distalmost part of the gonozooid [44]. In our material, this part was filled by a multicomponent coenocytic ‘cap’ (the upper part of the nutritive tissue) showing ultrastructural evidence of embryonic nourishment and also possibly isolating the gonozooid from inflowing sea water during larval release. Several larvae were situated beneath this ‘cap’, each surrounded by the cytoplasmic extensions of the nutritive tissue. We suggest that Borg was unable to see these very thin cytoplasmic ‘walls’ surrounding larvae in the upper part of the gonozooid in histological sections, and considered tightly appressed (but separate) cavities containing the mature larvae as a single “larval chamber”.

#### Pseudocoel and interzooidal pores

The presence of non-feeding gonozooids producing multiple larvae, indicates directional nutrient transport within cyclostome colonies. The question about its major route, i.e. interzooidal pores and their structure, remained unresolved. Harmer ([40], p. 213) was the first to state that “all the zooecia [zooids] are in organic connection by means of the funicular tissue, which passes through the pores from one zooecium to another, and from the zooecia to the ovicell [gonozooid].” Borg ([44], p. 228) followed Harmer, mentioning that the “strands of mesenchymatous tissue” in the pseudocoel are connected with the similar “irregular, sparse network” of the neighbouring zooids “by means of processes which pass through the interzooidal pores”. This contradicted another of Borg’s statements that the interzooidal pores serve for “exchange of coelomic fluid…and nutritive matter between various zoids” in cyclostomes, i.e. they are open ([44], p. 202; [81]).

This contradiction can be explained. The TEM-study of Nielsen and Pedersen [73] showed both open (distal) and occluded (basal) interzooidal pores in autozooids of *Crisia eburnea*. The authors agreed with Borg that interzooidal pores provide open communication between neighbour zooids, although some of them are closed at a later stage by either epithelial cells or by a calcareous plate (the latter based on an illustration by Boardman and Cheetham [82]). Moreover, Nielsen and Pedersen [73] found that closed interzooidal pores exhibited numerous internal spines and were filled by a single “cincture-cell” containing microfilaments, mitochondria, Golgi apparatus and scant RER—perfectly matching our observations. A similar structure of the closed interzooidal pores was also described by Carle and Ruppert [83], who studied *C. elongata*. In our material the ‘pore cells’ were sometimes in contact with processes of the ‘mesothelial’ cells of the pseudocoel (see also below).

SEM-study of the fine-scale skeletal structures in different cyclostome families by Taylor with co-authors showed that cyclostomes are characterized by spinelets developing inside the interzooidal pores and partially occluding them [84–88]. In some tubuliporine cyclostomes these internal spinelets were so numerous and tightly appressed (see e.g. [89]) that the pores should be clearly plugged by interspaced epithelial cells. Elsewhere, the pores in the young interzooidal walls of *Crisulipora occidentalis* are open, but later their lumen becomes gradually obstructed by calcified spines growing centripetally [80]. Comparing these observations with those of Nielsen and Pedersen [73] and our data, we suggest that such a narrowing of the pore lumen inevitably resulted in its occlusion by 1‒2 bordering epithelial cells (sandwiched between the pore spinelets). If so, the initial fluid (and nutrient) exchange between young neighbour zooids is substituted by the intracellular transport in the older ones, suggesting the structural and functional modification of the ‘pore cells’ for a transport function. The wide distribution of pore spinelets among cyclostomes (see above) could indicate that a similar situation is typical for this group, supposedly in both auto- and gonozooids. Thus, the interzooidal pore structure in cyclostomes is reminiscent of that in gymnolaemates rather than phylactolaemates, as Borg [44, 81] thought.

The cyclostome pseudocoel (exosaccal cavity) is a voluminous zooidal cavity that, according to the descriptions of early authors, contains so-called “funicular tissue” [40] or “strands of mesenchymatous tissue” ([43, 62]; [44], pp. 229–230). Borg [44] also described it as a network of “solitary mesenchymatous cells” with “long, thread-like processes”. Nielsen and Pedersen ([73], p. 66) briefly noted that the “network of ectodermal cells occupies the basal part of the cystid”. Moreover, in the studied crisiids, both Calvet [62] and Borg [44] described “leucocytes” and “amoebocytes”, correspondingly, in both the pseudocoel and zooidal coelom.

In contrast, our study on crisiids showed that the ‘mesothelial’ cells comprise a prominent element in the pseudocoel of both autozooids and gonozooids, forming a three-dimensional network with strongly varying structure and density. Its possible functions may be mechanical stabilization of the membranous sac position inside the cystid in autozooids, as well as an exchange of substances between pseudocoel and nutritive tissue in functioning gonozooids. Indirect evidence for this transport is the more numerous and physiologically active ‘mesothelial’ cells covering the membranous sac in gonozooids and contacting the nutritive tissue through cytoplasmic processes piercing the basal lamina. Similar processes are also formed by the tissue itself (Fig. 7c, d, see also above). The shape and ultrastructure of the cyclostome ‘mesothelial’ cells are reminiscent of the funicular cells in cheilostomes (e.g. [83]). In both cases, they are mostly elongated or irregular in shape and have an electron-lucent cytoplasm including a few lipid droplets and other organelles. Also, both may display signs of synthetic activity based on their RER cisternae and mitochondria [25].

The ‘mesothelial’ cells with homogeneous and striated paracrystalline inclusions we describe here are probably ‘storage’ cells, accumulating nutrient reserves as characteristic protein platelets (also detected in some larvae) (compare Figs. 7b,d, f, 8g and 10d). They resemble the nutrient-storage cells with homogeneous protein platelets found in the zooidal cavity of the placental cheilostome *Celleporella hyalina* ([25], see also [23, 24]). The frequent occurrence of such cells in the gonozooids of *Crisia eburnea* and *Crisiella producta* could reflect the necessity to store nutrients, ensuring against possible instability of nutrient transport from the autozooids. At least some of the “spherular leukocytes” depicted by Calvet [62] in *C. denticulata* could also be such ‘storage’ cells.

In both crisiid species examined, striated and homogeneous inclusions were also detected in some cells of the ‘upper cell complex’ in autozooids and gonozooids. Surprisingly, this massive cell structure was overlooked or neglected in all previous studies. The different ultrastructure of the constitent cells leads us to suppose that this ‘complex’ includes the ‘storage’, ‘synthetic’ and ‘totipotent’ (stem) cells. The latter could maintain the cell population in the pseudocoel, also participating in coenocytic ‘cap’ formation (see above).

### Gonozooid functioning

#### Gametogenesis and fertilization

In Crisiidae both spermatogonia and oogonia (the latter 5.4 μm in diameter, [43]) originate from the mesoderm (“funicular tissue” in [40]) in the growing zones (common buds) at the tips of branches, further differentiating to sperm or oocytes in gonochoristic zooids [40, 43, 44, 62, 66]. Male autozooids contain spermatogenic tissue, whereas most of the female germ cells degenerate; an ovary is established in a few developing autozooidal polymorphs (presumptive gonozooids) via association of the oogonium/early oocyte (normally one, but occasionally two per zooid) with the forming polypide bud. Since the oocyte is associated with the apical (lower) part of the polypide bud, growth of the latter results in the oocyte descending to the bottom of the forming zooid. The oocyte becomes surrounded by the follicle cells of mesodermal origin (“egg-follicle” or “primary follicle” of Borg [44]) and, together with the polypide, is enclosed inside the membranous sac. Our study in the White Sea did not sample the onset of reproduction in the local crisiid population because, as with some other bryozoans studied [90–92], it starts in late spring under ice. Nonetheless, in *Crisiella producta* we were able, apart of zooids with ovary, to detect a few zooids with early oocytes (20 μm in diameter) unconnected to the polypide and presumably destined to degenerate [44]. We think that such zooids grow to form normal autozooids. Similarly, in the ctenostome bryozoan *Alcyonidium* sp., Faulkner [93] described the origin of the germ cells from totipotent ones in zooidal buds, followed by ovary formation in prospect female zooids and degeneration in others.

Ovarian structure is simple. One, rarely two, small oligolecithal oocytes are surrounded by a single layer of flattened and, sometimes, cubic follicle cells ([44, 66]; our data). The diameter of the mature oocytes reportedly varies from 15 μm [44] to 25 × 20 µm (our data) in *Crisiella producta*, and from 17.6 μm [40] to 21.6 μm [43] in *Crisia eburnea*. Similar to all other incubating bryozoans [16, 94], fertilization is intraovarian: the male pronucleus was reportedly found in the mature oocyte in *C. producta* by Borg [44] who also stated that, at the next stage, the ovary is separated from the modified polypide, and the oocyte (upon reaching its definitive size) starts to cleave inside the follicle. This triggers quick degeneration of the polypide, but the membranous sac persists.

Harmer [40] and Robertson [43] assumed that the formation of the cyclostome gonozooid is triggered by the establishment of a connection between the oocyte and the polypide bud. We agree with this suggestion, and it seems that association of the early oogonium/oocyte with a polypide bud and establishment of the ovary results in polypide modification and, thus, shift in the developmental trajectory of the zooidal bud towards an autozooidal polymorph (prospective gonozooid). Borg ([44], p. 417) stressed that the establishment of the ovary results in a “peculiar transformation of the fertile polypide”.

In addition, Harmer [40] suggested, although indirectly, that failure of normal gonozooid development and functioning could be explained by failed fertilization. Considering the idea of fertilization being the main reason for gonozooid establishment, Borg [44] rejected this stressing that it would be inconsistent with the specific position of the gonozooid(s) in a colony and contradicts the limited number of gonozooids despite massive sperm production [31, 40, 43, 44, 66]. At the same time, sperm limitation could be the reason why only one gonozooid per colony is developed in many cyclostomes and many full-grown colonies lack them entirely (e.g. [95]). Moreover, experiments have shown that gonozooid development begins but fails in the absence of allosperm in the crisiid *Filicrisia geniculata* [69] corroborating the suggestions of Harmer [40].

In contrast, experimental work on a cheilostome bryozoan and one colonial ascidian demonstrated that sperm limitation is unlikely for aquatic invertebrates whose eggs are retained within the maternal body [96]. Accordingly, potentially all autozooidal polymorphs with established ovaries could be fertilized, but not all will become gonozooids. Borg [44] suggested that in some colony areas the autozooids might not provide sufficient “nutritive conditions” for development and functioning of multiple gonozooids. Further developing the idea of colony control over gonozooid development and energy limitation [36, 97], it is possible to assume that forming gonozooid(s) that established earlier or in some ‘key loci’ (i.e. having most effective position to get nutrients from autozooids) could possibly affect younger neighbouring polymorphs, suppressing their development into gonozooids. Taking together, this explains patterns and limitations of colony total resource allocation [90] and specific position of gonozooids, because each functioning gonozooid requires the input of multiple feeding zooids [40, 44, 70, 97]. On the other hand, life at great depth, strong currents and few available substrates could make sperm limitation important. We conclude that both sperm limitation [98] and colony control may play roles in different situations, and that it is premature to assign a single factor to gonozooid development.

#### Nutritive tissue: origin and functions

According to early descriptions, zygote cleavage inside the female gonad is accompanied by disintegration of the “primary” [i.e. ovarian] and formation of the “secondary” follicle [44]—a multilayered structure of large squamous cells that Robertson [43] simply described as a “follicle”. Distinct ‘layers’ of multiple nuclei in the “multinucleated follicle surrounding the egg” and early primary embryo were also depicted by Harmer ([40], pp. 199‒200, Pl. 22, Fig. 5). According to Borg ([44], pp. 421‒422) some constituted cells of the ovarian follicle migrate between blastomeres (denied by Dolinina [66]), whereas others disintegrate, and a massive “secondary follicle” (our multilayered ‘envelope’) is formed around the early primary embryo via continuous cell ‘detachment’ from the ‘inner [cell] layer of the membranous sac’. This activity decreases at some point, and progressively fewer cells are added to the ‘envelope’, whereas few others migrate between blastomeres. Finally, the “secondary follicle” is substituted by (i.e. transformed into) the “syncytium” with “irregularly scattered … numerous small nuclei”, i.e. “nutritive tissue”. Borg ([44], p. 427) confusingly concluded that the membranous “sac gives origin to all nutritive tissue found in the gonozooid except the trifting portion of it that originates from the primary follicle (see also below)”.

Our data confirm that the simple ovarian follicle (Fig. 5a) is substituted by a multilayered cellular ‘envelope’ developing around the embryo (Figs. 5b, 8a, b), although its origin is still uncertain (see also below). As the primary embryo grows, the ‘envelope’ around it gradually transforms into the conglomerate of mono- and binuclear cells and coenocytes (Fig. 8c, d). This recalls Robertson ([43], p. 136), who described multiplication of the “small follicle cells” close to the early embryo “accompanied by a diminution in number of the cells of the concentric layers” and suggested that the former are derived from the latter as “a stage in their absorption”. Bi- and multinucleated cells also appeared in the wall of the membranous sac (Fig. 8e), which becomes incorporated into the ‘envelope’. Finally, both the multicellular ‘envelope’ and its enveloping part of the membranous sac are replaced by the multinuclear ‘mass’, which lacks distinct membrane boundaries within. It is potentially possible that the coenocytes formed from the cells of both the multilayered ‘envelope’ and the membranous sac could merge (thus, yielding a true syncytium), whereas some cells/coenocytes are resorbed, leaving ‘gaps’ visible as large empty spaces in our preparations (Fig. 6a, inserts). Most likely, this precise stage was named a “vacuolated follicle” by Harmer [40], who described it as transitional during its transformation to the “protoplasmic reticulum [nutritive tissue]”.

The exact sources of the nutritive tissue remain unclear. In our TEM-images the wall of non-altered membranous sac consisting of the single cell layer was either adjoined (Fig. 8b), partly contacted (Fig. 8c) or free from the fully-formed multilayered cell ‘envelope’ (Fig. 8d). Accordingly, the latter potentially could be formed via dedifferentiation of some cells of the membranous sac followed by their divisions and displacement (see [44, 66]). Later, some such cells of the ‘envelope’ could further participate in the formation of the coenocytic elements. Some cells of the membranous sac wall also become multinucleated and apparently transformed directly to coenocytes (Fig. 8e).

In addition, at least some of the ovarian (follicle) cells also could potentially undergo dedifferentiation and divisions, helping form the multilayered ‘envelope’ and coenocytes. Either way, the membranous sac wall (initially, cellular and later coenocytic) is incorporated into the forming nutritive tissue. Our scenarios therefore correspond to the opinion of Borg ([44], p. 425) that “the nutritive tissue derived from the primary and secondary follicles” [the latter originating from the membranous sac]. Nonetheless, it remains unclear from Borg’s words how the ovarian (“primary”) follicle could participate in this process because he repeatedly mentioned its total disintegration.

Thus, the early nutritive tissue surrounding the embryo should originate from coenocytes developing exclusively from cells of the membranous sac wall, or from these together with the cells of the ovarian follicle. Since we could not detect the membrane boundaries inside the early nutritive tissue, we suggest that its coenocytic elements fuse, forming a syncytium. Another possibility involves a substitution of the multilayered ‘envelope’ by a single coenocyte, a scenario we consider less probable. In any event, this situation is remarkable because coenocytes are widely distributed among plants [99–101] and fungi [102], but known in animals only as temporary structures in the early development of some cnidarians, arthropods and teleost fishes ([103–106]; discussed in [75]) and the parasitic plasmodial stage in orthonectids [107; G. Slyusarev, personal communication] and myxozoans [108]. We should stress that the latter two cases convergently mirror situation in cyclostome bryozoans in having a multinucleated (plasmodial) ‘envelope’ that encloses and nourises multiple multicellular offspring (embryos and pansporoblasts). Besides, we are not aware of syncytia originating from coenocytes in any organism.

Another source of nutritive tissue is cells/coenocytes originating from the roof of the membranous sac (see above) in the distal part of gonozooid. The relative contribution of the different cell sources to the definitive placental analogue requires further study. An important aspect is the preservation of the basal lamina of the membranous sac. This indicates structural and developmental continuity between it and the placental analogue, with a corresponding shift in function from a mechanical (hydrostatic) to nutritive one (discussed below). In addition, the solitary cells (some binucleated) found within mature nutritive tissue could be the ‘stem’ cells providing growth and renewal of the placental analogue throughout gonozooid formation and functioning. At least some of the “giant-cells” described by Harmer [40] and Robertson [43] inside the nutritive tissue could be such stem cells. Borg [44] thought that the ‘giant-cells’ participate in the formation of this tissue in tubuliporid cyclostomes.

In summary, the composition of the fully-formed placental analogue is a complex mosaic in Crisiidae. Its proximal part (enveloping the primary embryo) develops as either purely coenocytically or coenocytic-syncytially from one or, possibly, two cell sources (membranous sac and ovarian follicle). The distal part is coenocytic, and originates from the membranous sac and the ‘upper cell complex’. The remaining nutritive tissue is structurally represented by coenocytes and large solitary cells, both presumably originated from modified cells of the membranous sac wall. Potentially, some coenocytic ‘elements’ could be syncytial there.

Interestingly, the early nutritive tissue seems to be more synthetically active than mono- (often with lobate nuclei) or binucleated cells of the multilayered ‘envelope’. This probably reflects their different functions: predominantly nutrient provisioning versus simultaneous provisioning and formation of the nutritive tissue. Similarly, the contrasting ultrastructure of different coenocytic ‘elements’ constituting the fully-formed placenta seems to reflect differences in their age and/or functional specialization. Some parts of the nutritive tissue clearly degrade. Embryonic growth and signs of endocytosis (e.g. Fig. 9b, d, g, h) unambiguously point to the nourishing function of the placental analogue, although the ultrastructure of certain areas shows that they are not involved in active synthesis. In contrast with the areas with well-developed RER and numerous free ribosomes and mitochondria, large parts of the placental analogue have far fewer such organelles. Both variants of the coenocytic ‘elements’ had multivesicular-like bodies and autophagosomes (sometimes numerous) of different size and with different content, indicating active rearrangement/reparation. Moreover, the arrangement, size, number and shape of the nuclei indicate that their divisions continue through the coenocytes, pointing to constant growth. The synthetically ‘non-active’ areas are potentially ‘old’ zones ‘recovering’ after an active period of functioning. Another interpretation is that these areas are predominantly involved in the bi-directional transport of substances between developing embryos and the nutritive tissue, supported by the nutrients accumulating in the pseudocoel. Because the peripheral zone of the nutritive tissue only rarely showed signs of endocytosis (Fig. 11a, insert), we suggest that it obtains most of the low-molecular precursors of nutrients from the pseudocoel by facilitated diffusion and active transport. Intercellular transport via appendages of the ‘mesothelial’ cells piercing the basal membrane should be also considered. The placenta accepts (and partly transforms) nutrients, further transporting them to the developing offspring. The cytoplasmic bridges between the adjoining coenocytic ‘elements’ could provide the necessary transport and redistribution of gases/nutrients within the entire placenta, whereas various cell contacts could guarantee the isolation of the embryonic ‘chambers/cavities’ for more effective nourishment—a general trend in the evolution of matrotrophy (see [7]). Removal of waste products from developing offspring should not be neglected.

### Brief comparison with other Cyclostomata

Among the main differences in the sexual reproduction between cyclostome families are the number, shape, position and development of gonozooids (reviewed in [31, 44, 109]; see also [95, 110]). In most cases each gonozooid is a modified autozooid, whereas in the family Lichenoporidae a common incubation chamber is formed inside the colony via resorption of internal calcareous walls and extension of the membranous sac(s), and nutritive tissue(s) from the fertile zooid(s) to growing incubation cavity [36, 41, 44]. The few differences from crisiids are as follows. Hermaphroditic zooids were described in Lichenoporidae [41]. The number of oocytes associated with a polypide in a presumptive gonozooid is normally one, but may reach six, and at least two primary embryos were detected in one gonozooid, each in a separate follicle [41, 42, 44, 48, 62]. In tubuliporids and lichenoporids the gonozooids begin their development as autozooids with a functional polypide that degenerates upon the onset of embryogenesis. Development and structure of the nutritive tissue are similar to crisiids, although for most groups the data is incomplete. The fission of secondary embryos into two parts giving rise to the tertiary embryos has been recorded in some tubuliporid, lichenoporid and hornerid cyclostomes [41, 42, 44], a feature that was reported first by Robertson [43] for one crisiid species. This phenomenon requires additional study.

The only documented extant cyclostome family without gonozooids is Cinctiporidae. Recently, small, although macrolecithal, oocytes were discovered in the pharyngeal wall of *Cinctipora elegans*. Compared to the large-sized ancestrulae, this suggests considerable embryonic growth and, thus, placentation. Incubation presumably occurs inside very large non-modified autozooids that potentially could hold multiple embryos, i.e. support polyembryony as in other cyclostomes [54].

### Embryonic development

#### Early embryogenesis

Harmer [40] was the first to note strongly modified cyclostome development. Before his discovery of polyembryony, it was thought that each embryo developed from a separate egg. The first illustrated description of embryogenesis was by Smitt [57], who depicted early secondary embryos consisting of identical cells constituting, however, the peripheral layer and central mass in *Crisia eburnea*. Barrois [58] correctly described the early (primary) embryo as morula and gave a good description of the larval anatomy and morphogenesis, although he thought that the initial stage in the formation of the adhesive organ is a gastrulation. This work was followed by Metschnikoff [59], who described bilayered (secondary) embryos as diblastulae (considering the inner layer as entoderm) and the stage with invaginating adhesive organ as pseudogastrula (see also [61]).

Harmer ([40], p. 215), who discovered primary and secondary embryos in crisiids, described the early cleavage as asynchronous, yielding a scattered group of blastomeres that later “come together” to form “a small rounded mass of undifferentiated embryonic cells [i.e. morula] …without any…definite layers”. The late primary embryo was confusingly described as either having an “ectodermic layer… clearly differentiated” or “obscurely differentiated onto outer and inner cells” (p. 209). Finally, when embryonic fission begins, the early secondary embryos were reported to inherit the bilayered structure. This view (possibly influenced by the earlier embryological works mentioned above and further confused by mentioning the “irregular cavity” of the primary embryo, p. 209) was supported by most researchers [44, 62, 65, 66, 111] and was included in text-books (e.g. [36, 72]). In fact, the “irregular cavity” in the distal part of the primary embryo described by Harmer [40] is constituted by the deep slits separating future secondary embryos (Table XXIII, Fig. 11). This author neither mentioned nor depicted any inner cavity inside the primary embryo. The same holds true for Calvet [62, p. 335], who only described the “central cavity” in the secondary embryos. In contrast, Borg [44, p. 422] wrote about “a small elongated cavity…” as being a “part of a greater, irregular cavity in the interior of the primary embryo”.

Our data on embryonic structure and development differ substantially from these descriptions, since neither primary nor early secondary embryos exhibited distinct layers or cavity in the material studied (Fig. 6a, insert). Analysis of Harmer’s [40–42] illustrations showed no signs of a bilayered structure. The only indication is the early embryo depicted in [40], Pl. 23, Fig. 8, showing a darker periphery. We saw similar staining in a few of our images (e.g. Fig. 5e), but the external and internal embryonic cells were otherwise identical. Robertson ([43], p. 141) also wrote that “neither in the primary embryo nor in the buds when first set free, is there any differentiation into cell layers… No cavity is present” in *C. eburnea* (also stated by Nielsen [64]).

In accordance with that, the early primary embryos had a loose and irregular cell arrangement, and resorption of some blastomeres was not uncommon during embryogenesis in our histological sections. The latter fact could explain the statements of the early authors about the “scattered blastomeres”, “free” blastomeres and “the separation of blastomeres and the interpolation of the follicle cells” during the earliest cleavage stages ([40], p. 215; [43], p. 136; [44], p. 422). This situation requires checking, e.g. a similar phenomenon was described in some flatworms with complex eggs [112] and in salps [113]. Formation of the secondary embryos occurred without formation of the finger-like processes but rather via detachment of the rounded/oval ‘lobes’ from the irregular-shaped late primary embryo (Figs. 5f, 6b, insert, c). Both early primary and early secondary embryos were compact morulae, and a bilayered structure via delamination and central cavity appeared later in the secondary embryos. Differences in descriptions may be explained by rough fixation methods applied by previous researchers or, possibly, by misinterpretation of the images as the blastomeres in the early primary embryo are often not clearly distinguished.

#### Extraembryonic nutrition

Harmer ([40], p. 221) precisely indicated that large primary embryo and multiple larvae could develop from the small-sized egg only if the “nutrient material” is transported via pores from zooids with functional polypides. He wrote that “nutriment is conveyed to the developing larvae” by means of the “rich protoplasmic reticulum” and suggested that the secondary embryos inside its large “vacuoles” consume the “albuminous fluid” by “diffusion”. Interestingly, Borg [44] thought that the secondary embryos used cells of the ovarian follicle and some cells of the multilayered ‘envelope’ migrating between blastomeres as nutritive material.

Cells of the primary embryo show no signs of endocytosis on our TEM-images. Some of them, however, contain small lipid droplets in their near-surface cytoplasm which are similar to those in the surrounding early nutritive tissue (Fig. 8c, d). This suggests some nutrient transport from the placental analogue to the developing embryo via, e.g. facilitated diffusion and active transport. In contrast, small pits and vesicles associated with the apical plasma membrane in the peripheral cells of the early secondary embryos indicate endocytosis. Although no direct signs of exocytosis were detected on the adjoining membrane of the nutritive tissue, that membrane was convoluted in some areas, suggesting such an opportunity (Fig. 9b, insert, d).

Early larvae with an extensive microvillous ‘cover’ developing simultaneously with ciliary corona point to considerable enlargement of the larval surface and, thus, intense nutrient consumption. Studies on mammalian gestation have demonstrated the importance of the surface area through which nutrient transfer occurs [4, 114, 115]. Moreover, numerous pinocytotic canals and vesicles between the bases of the microvilli were detected (Fig. 9f‒h), indicating another mechanism of extraembryonic nutrition. Both secondary embryos and early larvae consume nutrients all around, as was previously shown for most of the gymnolaemates studied ([15, 25, 26], but see [116]).

Our data on the microanatomy of early larvae generally correspond to the description by Nielsen ([64], p. 223) based on histological sections of *Crisia eburnea* and *Crisiella producta* (see also [68]). Among other details, he mentioned cells with numerous “spherical bodies of unknown significance” encountered in different cell layers. We suppose these to be reserve nutrient granules accumulated by the larvae during matrotrophic incubation, and visible due to their size and paracrystalline structure. Cells of the adhesive organ on our TEM-images resemble those on the TEM-microphotographs of the early larvae of *C. denticulata* made by d’Hondt [67].

### Evolution of placentation among Bryozoa

In aquatic invertebrates and chordates the multiple shifts from a broadcasting (free release of both ova and sperm into the water column resulting in external fertilization) to a spermcasting strategy (only sperm is released, followed by internal fertilization) were a prerequisite for acquiring parental care in the form of offspring retention—either preparitive (viviparity) or post-paritive (brooding) [5, 16, 20, 117]. Both variants were accompanied by the independent evolution of matrotrophy, in particular, placentotrophy [6, 7].

Among modular suspension feeders, placental analogues are known in a few sponges and entoprocts, some colonial ascidians, many bryozoans and all salps. Placentotrophy is also suggested in some acroporid corals (reviewed in [7]). While the most complex placental analogues are known in Salpidae ([118], see also below), bryozoans show a much greater positional and developmental diversity in this respect, with placentas originating at least 23 times in all three classes ([7, 17]; see also [18]). In contrast to the majority of placental bryozoans that are brooders (all phylactolaemates and most gymnolaemates), all Cyclostomata and the small gymnolaemate family Epistomiidae are viviparous [16, 19]. Phylactolaemata brood their embryos in an internal brood sac (reviewed in [20, 119, submitted]), as do some placental ctenostome and cheilostome Gymnolaemata (reviewed in [16, 36, 120]). Some ctenostomes also brood their progeny in the tentacle sheath modified into a placental analogue (reviewed in [26]). Finally, most placental cheilostomes incubate their young in skeletal brood chambers (ovicells) (reviewed in [16, 19]). In all these cases, the brood cavity is external with respect to the body cavity (e.g. [121, 122]), and cleavage starts after oviposition of a zygote from the maternal coelom to the brood chamber via the coelomopore (reviewed in [79]). The placental analogues are formed by modification of the epithelium of the body wall—cystid wall, ooecial vesicle or tentacle sheath, whose function is shifted from mechanical/protective to nourishing. Notably, in all matrotrophic cheilostome brooders, cells of the placental analogue are separated from the embryo by the body wall cuticle, which is permeable for nutrients [15, 16, 23–25, 78].

In contrast, in cyclostomes embryogenesis begins in the ovary and continues inside nutritive tissue that substitutes for the coelomic cavity. In the cheilostome family Epistomiidae a single embryo develops intracoelomically, and the placental analogue develops from either the follicle (ovarian) or nurse-cells [22, 123, 124]. In cyclostomes the major source of placenta is cells derived from the membranous sac (modified peritoneal lining) and, possibly, the ovarian wall ([40, 44]; our data).

The dynamics of placentation is also quite contrasting: in most cheilostomes and some ctenostomes, placental analogues function without affecting the polypide (whether ordinary or rudimentary) recycling: they develop repeatedly for each embryo and degrade after larval release. In contrast, in ctenostomes nourishing embryos in the modified tentacle sheath, the polypide degenerates before or after deposition of a zygote in the brooding site (tentacle sheath, brooding pouch) [16, 26, 120, 125]. In Epistomiidae, the polypide degenerates irreversibly in the enlarged/swollen female zooid that produces a single larva [22]. Similarly, in cyclostome bryozoans the placental analogue develops once, being accompanied by polypide degradation and (gono)zooidal enlargement, but the embryos are numerous. Finally, the structure of the placental analogues in Gymno- and Phylactolaemata differs drastically from those in Stenolaemata, being cellular and coenocytic (and, possibly, syncytial), respectively. Their development therefore differs as well: cellular multiplication and hypertrophy versus nuclear multiplication and cytoplasmic growth.

Despite numerous differences, ultrastructural studies of placentae indicate common cytological mechanisms in their functioning. In the placental species of all three bryozoan classes, Gymnolaemata, Phylactolaemata and Stenolaemata, mature placental analogues show an active synthetic apparatus possessing numerous ribosomes and mitochondria and developed RER ([15, 24–26, 116, 119, *submitted*]). High transport and secretory activities are demonstrated by developed Golgi apparatus and abundant vesicles as well as by the microvillous or folded appearance of the apical membranes facing the embryos. Moreover, early larvae show similar trophic modifications in all cases. Their surface cells bear developed microvilli or a network of irregular projections and show signs of endocytosis (pinocytotic canals and small vesicles below the cell membrane, sometimes coated pits) ([15, 25, 26, 116, 119, *submitted*]). As a result of this functional convergence, placental analogues having a different positional, developmental and morphological basis in the representatives of phylogenetically distant groups, all provide bidirectional transport of substances. In this respect bryozoan placentation is especially remarkable, being an excellent example of exaptation—features that acquired functions for which they were not originally adapted [126]. Various bryozoan brood chambers evolved as embryonic protective organs that additionally became the sites of their nourishment. In some cases, however, embryos are incubated inside structures (eversible tentacle sheath in ctenostomes, membranous sac in cyclostomes) that normally participate in polypide protrusion and retraction, but ultimately became the nutritive organs in both evolution and ontogeny. This phenomenon can also be described in terms of a ‘substitution of function’ accompanied by the corresponding change in structure.

Functional (and, correspondingly, structural) changes of organs and organ systems in evolution were exhaustively described and classified by a number of authors ([127–131]; reviewed in [132–136]). Using predominantly examples among vertebrates, they aimed to formulate the major principles of functional evolution. In cyclostome bryozoans, the hydrostatic function of the membranous sac changes to the nutritional one in both ontogenesis and phylogenesis that can be considered first as an example of substitution, both morphological [128] and physiological [131, 132, 134]. The substituting organ (nutritive tissue) functionally and structurally replaces the substituted one (membranous sac wall). During this process some elements of the sac resist (basal membrane), some disappear (ring muscles and probably some epithelial cells) whereas others are modified (the rest of the epithelial cells). This situation could be generally compared, for instance, with that in brooding ophiuroids incubating their offspring inside the bursae which original functions in non-brooding relatives are respiration, excretion and gamete release. In matrotrophic species the walls of the bursae seemingly provide nourishment for the developing larvae (reviewed in [7, 137]).

Both these examples could be also considered as an extension of functions—obtaining of additional (secondary) functions [129]. Some bivalve mollusks brood their larvae in the specific areas of their gills, and incubation is accompanied by a reduction of water circulation in them and, sometimes, acquisition of extraembryonic nutrition (reviewed in [7]). Thus, the main (primary) function is temporarily lost during incubation. In cyclostome gonozooids the hydrostatic function is lost irreversibly.

In this context, development of cyclostome nutritive tissue could be also described as a function change [127] as weakening of the main or strengthening of an additional function that results in the restructuring of the organ morphology and a dominance of secondary function while the initial main function either becomes secondary or disappears. Change of function is one of the most common principles of functional evolution including such iconic examples as transformation of the insect ovipositor to a sting and walking limbs into flippers. Nowadays the principles of the functional substitution have further developed being widely applied to the patterns of molecular evolution (reviewed in [138–141]).

### Evolution of cyclostome reproductive pattern

Evolution of matrotrophy triggered a shift in resource allocation during offspring development in both vertebrates and invertebrates: the higher degree of extraembryonic nutrition correlates with less intense vitellogenesis (e.g. [19, 142] and references therein). As to bryozoans, it was Harmer [40] who compared cyclostome reproduction with that in placental mammals, both having combination of small, yolk-poor eggs and extra-vitelline nourishment. It was suggested earlier that the emergence of placentae resulted in a shift from macro- to oligolecithal oogenesis in both gymnolaemate groups—cheilo- and ctenostomes [15–17, 19]. In both cases, an initial step was presumably the shift from the production of numerous yolk-poor, small oocytes, a zygote-spawning strategy and non-incubated planktotrophic larva to macrolecithal oogenesis accompanied by the reduction in oocyte number, evolution of brooding and lecithotrophic larva. The acquisition of a placenta resulted in a reversal to oligolecithal oogenesis accompanied by production of fewer oocytes.

As to cyclostome bryozoans, we propose two hypothetical scenarios. Numerous female germ cells developing in the budding zone of modern Cyclostomata [43, 44] is clearly an ancestral feature, but the number of oogonia/oocytes associated with the polypide-bud is much less (1‒6), and only 1–2 oocytes develop to an embryo that possibly is a secondary trait (reviewed in [16]). If an ovary initially produced many small oocytes (as in some zygote-spawning gymnolaemates), the way to reduce their number could be by an early start of cleavage directly inside the ovary (among bryozoans also known in epistomiid cheilostomes [22] as well as in some matrotrophic cnidarians, gastropods, nemerteans, arthropods, echinoderms [7] and teleost fishes [143]). This would suppress further divisions of oogonia and oocytic production. The transition to intraovarian embryonic development (i.e. viviparity) potentially could meet the problem of gas exchange and waste removal, i.e. transport of substances from the parent to embryos and back. This condition could have given rise to the origin of placentation via transformation of the membranous sac (and, possibly, follicle). While the extraembryonic input to the developing progeny was small, the number of incubating zooids in a colony was probably large as occurs in gymnolaemates. Embryos were incubated in non-modified autozooids and released, depending on nutrient provisioning, as either feeding or non-feeding larvae. Evolution of substantial placentation could result in non-feeding larva and yield polyembryony in enlarged gonozooids [19], and a corresponding reduction in gonozooid number to a few or just one to fit the colony energy balance (see also below). In Cinctiporidae gonozooids were lost as autozooidal size drastically increased. The eggs became macrolecithal although remained small in this clade [54].

The second scenario involves an initial shift from oligo- to macrolecithal oogenesis accompanied by a reduced number of oocytes and acquision of non-feeding larva (similar to gymnolaemates). Macrolecithal oocytes of *Cinctipora elegans* are apparently produced in the pharyngeal wall, but this comparison could be relevant since the ovary is connected to the polypide in other cyclostomes too. Whether oogenesis may shift to macrolecithal mode in some ancient cyclostomes is unknown. In any case, the subsequent steps could be the same as in the first scenario: starting from intraovarian cleavage and acquisition of placental analogue, resulting in polyembryony and the evolution of gonozooids. The origin of placentation, however, ‘returned’ oogenesis to the oligolecithal mode. In cinctiporids macrolecithal mode retained, however, in similar to some placental gymnolaemates [16, 17, 19]. These hypotheses could be potentially tested by plotting data on oogonesis in various cyclostome lineages against phylogeny, but currently insufficient data are available for this work.

### Is the cyclostome placenta unique?

Coenocyte placental analogues of cyclostome bryozoans are the structural and functional (but not developmental) equivalent of the syncytial placentae known only in a few invertebrate phyla. Among platyhelminthes, syncytiality is characteristic of the surface epithelium as well as the epithelium of the excretory and reproductive ducts of Cestoda (reviewed in [144, 145]). Placentation has repeatedly evolved in the phylogenetically distant cestodes via apposition of the developing embryos to the uterine syncytium, which forms multilayered cytoplasmic outgrowths enveloping individual embryos. In all studied species with obligatory placentation, the uterine syncytium shows signs of intense protein synthesis and secretion (reviewed in [146–148] and references therein). This ‘embedding’ of embryos in nourishing syncytial elements is strongly reminiscent of the situation in cyclostome bryozoans.

In the monogenean family Gyrodactylidae the external tegument, the absorptive layer of the intestine and some parts of reproductive system, including the uterus, have a syncytial structure [149, 150]. In the matrotrophic gyrodactylids, the surface of embryonic cells adjacent to the uterus wall shows signs of endocytosis [151]. This resembles the cyclostome secondary embryos absorbing nutrients all over their surface during incubation. Besides, unlike most described placental analogues (see above), neither the uterine syncytium of gyrodactylids nor large parts of the cyclostome nutritive syncytium showed prominent synthetic activity. Rather, their ultrastructure indicates active nutrient transport (transcytosis) (e.g. [149, 152]; our data). Finally, the basal lamina of the gyrodactylid uterine lining is thought to participate in nutrient and waste transport as a filter for macromolecules [149, 152], which might also be the case for the basal lamina of the membranous sac in cyclostome gonozooids.

In almost the entirely matrotrophic onychophoran family Peripatidae, the syncytial placenta is formed from uterine epithelium at the site where the embryo is attached to the uterine wall and further spreading over most of the uterus as the embryo grows. This syncytium has a microvillous cover on both sides, presumably acquiring nutrients from the maternal haemocoel and transporting them to the embryo cavity via the adjacent embryo sac wall; this would make them analogues to the mammalian noninvasive epitheliochorial placenta [153–155]. The attachment of the embryo to the uterine wall presumably induces formation of the placental syncytium. This is comparable to the early formation of coenocytic elements by the membranous sac in cyclostome bryozoans (triggered by the onset of cleavage).

All salps are placental [156, 157]. The early placenta is of maternal origin, deriving from a knob of follicle cells [113, 158]. While growing it merges into the syncytium and fuses with a modified part of the maternal blastozooid epithelium [157, 159]. Similarly, the cyclostome zygote starts cleavage within the follicle, which possibly participates in forming the placental analogue. The mature placenta of salps consists of two syncytial layers of maternal origin (the cortical one being secondarily added by embryonic leucocytes). These are closely connected by interdigitating microvilli [157], resembling the coenocytic ‘cap’ developing from the ‘upper cell complex’ and the membranous sac in crisiids. As in cyclostome coenocytes, the placental syncytia of salps contain numerous mitochondria, vesicles with material of varying electron density and RER cisternae [157]. This indicates both synthetic and transport activity.

Finally, among vertebrates the hybrid fetomaternal syncytial structures are characteristic of all studied ruminants, lagomorphs and marsupial *Perameles*. Among carnivores and bats with endotheliochorial placenta, the maternal uterine epithelium is either cellular or syncytial, being further displaced by fetal syncytiotrophoblasts (reviewed in [4]). A syncytial placenta of solely maternal origin is known in skinks of the genus *Mabuya* [160, 161]. Importantly, all aforementioned syncytial placentae are initially cellular, becoming syncytial via cell fusion. In contrast, the nutritive tissue of cyclostome bryozoans mostly originates via multiplication of nuclei and cytoplasmic growth, although coenocytic fusion is potentially possible.

Another important difference is that, despite the functional commonalities (nutrient synthesis and transport plus gas exchange and waste removal), all above-listed placentae develop as derivatives of sexual organs—modified uterine epithelium or ovary. In contrast, the bryozoan placental analogues evolved as a result of functional substitution, mostly involving no connection with the reproductive system. For instance, the nutritive tissue of Stenolaemata, with a possible contribution by follicular cells, is mainly formed anew at the basis of the zooidal hydrostatic system (the membranous sac). Similarly, in both placental gymnolaemates and phylactolaemates, embryo nutrition is provided by a hypertrophied epithelium of the maternal body wall (see above). From this perspective, this is reminiscent of the extensive network of excretory ducts associated with the uterus of the cestode *Skrjabinacanthus diplocoronatus*; this network distributes large quantities of lipids and is apparently involved in supplying nutrients to developing eggs [162]. A distant analogy can be drawn also to matrotrophic sponges, whose developing embryos are enveloped by the “nutritive follicle”, “placental membrane”, “epilarval trophocyte epithelium”, “nurse cells”, “nutrient capsule”, etc. comprised of modified cells of the mesohyl, choanocytes, pinacocytes or collencytes (reviewed in [7, 163] and references therein).

The occurrence of syncytial and coenocytic elements in both vertebrate and invertebrate placentas raises the question of their advantages. A syncytial/coenocyte structure could promote an effective exchange of substances because of the reduced number of membrane barriers while maintaining structural integrity. Besides, a syncytium/coenocyte is capable of rapid increase and has considerable structural plasticity [4]. This enables regional differentiation, which could be crucial in the case of multiple and asynchronous embryo development. Nevertheless, cellular placentae co-exist with syncytial ones, both performing successfully, as evident in various animal groups. To conclude, syncytial/coenocytic placentae evolved independently in different phyla, and in each case this seems to be an individual ‘solution’ based on either phylogenetic constrains, ‘developmental potential’ of a particular clade or particular circumstances arising during gestation.

### Polyembryony

The phenomenon of polyembryony—the development of two to several embryos from one egg—should be interpreted as the precocious asexual reproduction via fragmentation that begins at the early embryonic stage [164]. It should not be considered as budding, which always occurs from differentiated somatic cells. In a wider sense, it should be also viewed as a production of larvae (i.e. fully-formed autonomous, although non-adult, organisms) from non-equal clusters of blastomeres (embryos) via some regulatory mechanisms.

The prerequisites and consequences of polyembryony are intriguing evolutionary issues. The origin(s) of intracoelomic incubation (viviparity) in cyclostome bryozoans triggered an entire cascade of changes resulting in the acquisition of extraembryonic nutrition reflecting the active interaction between embryo and parent [19]. In turn, evolution of substantial matrotrophy might support the origin of polyembryony which is thought to be an integral stage in the life cycle and a synapomorphy of Cyclostomata [36, 88]. This phenomenon is rather rare in metazoans but is obligatory in at least 18 taxa from six phyla and has evolved at least 15 times; most of the cases involve parasites, with the exception of cyclostomes and armadillos [164–166]. The “unpopularity” of this reproductive mode is unsurprising given its shortcomings, primarily a reduced genetic diversity of the offspring as a result of the multiplication of a single genotype [97, 98, 165].

In the crisiids, however, the genetic variation among larvae from different gonozooids (i.e. potentially having different paternity) in the same colony may be sufficient for their survival [97, 167]. Furthermore, genetically identical larvae of cyclostomes are released sequentially over a lengthier period (> 2 months) [70]. This enables repeated testing of the same genotype against changing conditions and may be evolutionarily equal to sexual reproduction testing different genotypes against the same environment simultaneously [167].

The production of numerous descendants from one/few zygotes in a colony may compensate for limitations (e.g., phylogenetic) in egg number or the rarity of fertilization events ([98]; see also above). It may also serve as an alternative outcome of the trade-off between number and size of progeny. Polyembryony could be advantageous when brooding females are unable to store sperm, which may be the case in cyclostomes. More importantly, it potentially enables enlarging offspring number in response to increased food availability. For both endoparasites and sedentary suspension feeders, food abundance can change unpredictably. If, however, embryonic multiplication and growth is extended over time, offspring number would depend not on initial egg quantity but on the physiological condition of the parental organism during embryogenesis [165]. Moreover, placentation combined with polyembryony in cyclostomes may have the advantage of fast production of numerous non-feeding larvae that will establish new colonies during favorable periods. Based on published data on cheilostome bryozoans, the suggestion is that the duration of offspring development (oogenesis + embryogenesis) is shorter in placental versus non-placental brooders because embryonic development and growth occur simultaneously [16, 17, 19, 23]. This may be especially important in seasonal (i.e. less stable) environments, allowing faster occupation of vacant niches. In habitats with stable conditions (warm climate or deep-water) this would allow the completion of more reproductive cycles per year. Indeed, both the diversity and biomass of cyclostomes around New Zealand, the Antarctic, Mediterranean and in Arctic seas can be very impressive (e.g. [54, 95, 168–170]; Kotenko & Ostrovsky, personal observations). These explanations are applicable to both placental gymnolaemates (with numerous brooding zooids per colony) and cyclostomes (with one/few gonozooid(s) producing multiple larvae).

Grbic [166] concluded that, in parasitic insects, the evolution of all polyembryonic lineages included a transition from ecto- to endoparasitism, and the possibility to exploit the host’s nutritive environment for embryo development led to a change in egg size from large yolky to small. Similarly, the emergence of matrotrophy and polyembryony in cyclostome bryozoans might be connected to the shift towards oligolecithal oogenesis (see above). Another group sharing the unique combination of viviparity, placentation and obligate polyembryony with cyclostomes is armadillos* Dasypus*. In the latter, polyembryony is the only way to overcome the limitations in uterine shape and of a single implantation site, boosting the number of offspring [165, 171]. As in cyclostomes, however, polyembryony could not have occurred without a preexisting effective mechanism for supplying offspring with nutrients.

In summary, it is clear that massive larval production via polyembryony in Cyclostomata could not have evolved without the evolution of a highly advanced placenta. Cyclostome nutritive tissue is a highly integrated system able to quickly grow and rearrange/rejuvenate, effectively delivering and distributing nutrients to the numerous descendants. Extensive nourishment is directed from many feeding zooids to one or a few gonozooids that then serve as colonial ‘incubation organs’. This pattern of resource allocation contrasts to that in phylactolaemate and gymnolaemate bryozoans in which every incubation chamber is associated with an individual feeding zooid that is often the main source of nourishment. In our opinion, the massive provisioning of a very restricted number of ‘consumers’ was a crucial novelty that shaped the evolution of polyembryony. We suggest that quick multiplication of continuously growing offspring met a requirement to provide additional space for incubation, resulting in the evolution of cyclostome gonozooids.

### Conclusions

Gonozooid establishment in cyclostomes is probably governed by the general patterns of colony growth and resource allocation, although sperm limitation could also play a role in some cases. In Crisiidae transformation of zooidal bud to autozooidal polymorph is triggered by the association of the young oocyte(s) to a polypide bud whereas the autozooidal polymorph with modified polypide starts its development to a gonozooid after fertilization and intraovarian beginning of cleavage. Gonozooid formation includes a strong enlargement of the cystid and the degeneration of the polypide and its hydrostatic system, although the basal lamina of the membranous sac persists. The placental analogue is initially a multilayered cellular ‘envelope’ that is ultimately substituted by the coenocytic nutritive tissue. It has a complex origin, being formed from the cells of the membranous sac and, presumably, follicle cells, both becoming coenocytes. Fusion of the coenocytes into syncytial elements is potentially possible too. The fully-formed placental analogue of crisiids has a unique structure comprising a variety of coenocytic ‘elements’ of contrasting ultrastructure connected by cytoplasmic bridges and various cell contacts, and presumed ‘stem’ cells. Ultrastructural evidence demonstrates that the nutritive tissue is a highly integrated and actively rearranging system involved in both the synthesis and transport of nutrients to developing embryos and young larvae. Coenocytic placental analogues have never been previously described in animals.

Embryogenesis involves polyembryony. The primary embryo is initially a round morula that becomes irregularly lobed during growth. After separation, the lobes become secondary embryos without cell differentiation (with later delamination). Formation of the central cavity and organogenesis yields the larva. Both secondary embryos and larvae absorb nutrients via endocytosis and, probably, active transport and facilitated diffusion because early larvae bear microvilli that greatly increase the absorptive surface area.

Combining polyembryony with placentation yields many descendants that can potentially adjust their number to food availability for the maternal colony. Internal retention of embryos is a prerequisite for the origin of placental matrotrophy which, in turn, could stimulate the evolution of polyembryony and gonozooids in Cyclostomata. Moreover, oligolecithal oogenesis in cyclostomes might be a consequence of acquiring matrotrophy—a general trend in matrotrophs, both vertebrate and invertebrate. Despite clear limitations, e.g. reduced genetic diversity of the offspring and betting on an unverified genotype, polyembryony can be beneficial for cyclostomes. Production of numerous descendants from one to a few zygotes may compensate for fewer eggs although reduces the number of fertile zooids. A lengthier and sequential release of identical larvae enables repeated testing of the genotype against changing conditions and thus may be an evolutionary equivalent to sexual reproduction.

The complex coenocytic placenta of cyclostome bryozoans is comparable with the syncytial placentae of certain vertebrates and invertebrates. It shows similar structural and functional adaptations that enhance nutrient synthesis and trophic interactions with embryos, albeit differing in origin. Known placentae are mostly formed via modification of reproductive organs, sometimes with a contribution of embryonic cells. Nutritive tissue of cyclostomes, although possibly partly originating from the follicle cells, mostly develops from the membranous sac, which initially has a hydrostatic function. This example of functional substitution, along with certain other placental bryozoans, can be considered an exaptation. The coenocytic/syncytial organization of placentae may have some advantages such as simplified nutrient distribution (by reducing the number of membrane barriers), structural plasticity, rapid growth and integrity along with flexible synthetic and transport activities.

In the studied crisiids, the interzooidal pores bear numerous small internal spines and are filled by one–two specialized ‘pore cells’. This is presumably the basic mode in fully-grown cyclostome zooids. In the pseudocoel the irregular network of ‘mesothelial’ cells (more numerous and physiologically active in gonozooids) is probably responsible for maintenance of the membranous sac shape and position, nutrient accumulation and the intracellular exchange between pseudocoel and coelom/nutrient tissue. There is also evidence of a direct transfer of low-weight molecules via the basal lamina of the membranous sac, which may serve as a dynamic filter. We also interpret both ‘pore-cells and ‘mesothelial’ cells to participate in the intercellular transport of substances within a cyclostome zooids and colonies. The ‘upper cell complex’ is a possible source of totipotent cells for maintaining and restoring the cell population in the pseudocoel and also participates in the formation of the coenocytic ‘cap’ in gonozooids.

## Methods

Reproductive colonies of *Crisia eburnea* (Linnaeus, 1758) [172] and *Crisiella producta* (Smitt, 1865a) [56] (Figs. 1, 2) growing on kelps and red algae were collected during the ice-free period from 5 to 15 m depth by boat dredging and SCUBA-diving near the Educational and Research Station “Belomorskaia”, Saint Petersburg State University (Chupa Inlet, Kandalaksha Bay, White Sea). Sampling took place in June and August 2015, from June to October in 2016, in June 2017 and in August 2018.

Freshly collected colonies were fixed in 2.5% glutaraldehyde (buffered in 0.1 M Na-cacodylate buffer with 10.26% sucrose, pH 7.4) for 3 h and subsequently rinsed three times in the buffer. Postfixation was done in a 1% solution of osmium tetroxide (OsO4) in the buffer solution for 1 h followed by three rinses in the buffer. Decalcification involved several hours in 5% solution of EGTA in the buffer. After rinsing in the buffer, all colonies were dehydrated in a graded ethanol series (30–50–70–80–90–96%) and in acetone-resin mixtures (3:1–1:1–1:3) and subsequently embedded in epoxy resin (Agar LVR—Low Viscosity Resin). Resin blocks were sectioned using a Reichert UltraCut S microtome with Diatome Histo-Jumbo and Diatome 35 Ultra diamond knives (Diatome, Bern, Switzerland). Serial semithin (1.0 μm) and ultrathin (60 nm) sections were prepared for examination under light and transmission electron microscope (TEM), respectively. Semithin sections were stained with Richardson's stain; ultrathin ones were placed on copper grids and contrasted with 2.5% gadolinium triacetate and 3% lead citrate. Sections were examined using Zeiss Libra 120 transmission electron microscope (Zeiss, Jena, Germany) and photographed with a digital CCD Olympus Morada G2 (11 MP, in column) camera.

Some living and fixed colonies were photographed with a digital Leica DFC295 camera attached to a Leica M205C stereomicroscope. Altogether, we examined approximately 50 gonozooids from 30 colonies anatomically and ultrastructurally.

## Data Availability

The datasets analyzed during the current study are available from the corresponding author on reasonable request. All data needed are included in the paper.
